# Effect of injectable platelet rich fibrin augmented with vitamin c compared to injectable platelet rich fibrin alone on healing of necrotic mature single rooted teeth with chronic periapical periodontitis following regenerative endodontic therapy (a randomized controlled trial)

**DOI:** 10.1186/s12903-026-09924-w

**Published:** 2026-10-12

**Authors:** Wesam Osama Hassan, Ahmed S. ElSheshtawy, Manal Mohamed Abdelbaki, Geraldine M. Ahmed

**Affiliations:** 1 Department of Endodontics, Faculty of Dentistry, Cairo University, Cairo, Egypt; 2 Department of Endodontics, Faculty of Dentistry, Misr University for Science and Technology, Cairo, Egypt; 3 Faculty of Dentistry, Cairo University, Cairo, Egypt

**Keywords:** Regenerative endodontics, Pulp regeneration, Injectable platelet rich fibrin, i-PRF, Vitamin C, Ascorbic acid.

## Abstract

**Objectives:**

To assess the healing potential, postoperative pain and clinical normalcy associated with injectable platelet-rich fibrin combined with Vitamin C in regenerative endodontic procedure using cone-beam computed tomography and two-dimensional radiograph.

**Methods:**

Twenty-eight healthy patients with mature permanent single-rooted teeth diagnosed with pulp necrosis and chronic apical periodontitis were randomly allocated into two groups (*n* = 14). Regenerative endodontic treatment was performed using injectable platelet-rich fibrin combined with Vitamin C (intervention group) and injectable platelet-rich fibrin alone (control group). Periapical healing was assessed using CBCT and periapical radiographs over 12 months. Postoperative pain and clinical normalcy were also evaluated.

**Results:**

Both groups showed significant lesion reduction at 12 months (*P* = 0.001), with no significant between-group differences in postoperative lesion size (*P* = 0.981) or reduction magnitude (*P* = 0.497). CBCT-PAI scores improved in both groups without significant intergroup differences at baseline or follow-up. Two-dimensional radiographs showed a baseline difference (*P* = 0.007) but no postoperative differences; both groups demonstrated significant longitudinal reduction (*P* < 0.001). Postoperative pain scores were comparable between groups at all time points (*P* > 0.05), with a significant temporal decrease observed only in the intervention group (*P* = 0.001). All teeth remained asymptomatic during follow-up.

**Conclusions:**

Both i-PRF combined with vitamin C and i-PRF alone appear to be effective regenerative scaffolds, demonstrating comparable clinical and radiographic outcomes in regenerative endodontic procedures.

**Clinical trial registration:**

The study was registered with ClinicalTrials.gov (ID: NCT05585359), registered on 14 October 2022. Available at https://clinicaltrials.gov/study/NCT05585359.

**Supplementary Information:**

The online version contains supplementary material available at https://doi.org/10.1186/s12903-026-09924-w.

## Introduction

Periapical periodontitis is an inflammatory condition that develops secondary to pulpal infection, most commonly arising from dental caries or traumatic injury. Conventionally, this condition is managed by root canal treatment [1]. However, pulp extirpation may compromise the structural durability of endodontically treated teeth, rendering them more prone to fracture and reinfection [2]. These limitations underscore the essential role of the pulp tissue in preserving tooth vitality and long-term dental health.

Over the last decade, various endodontic and pediatric dentistry organizations have endorsed the regenerative procedure as a treatment modality for immature permanent teeth presenting with necrotic pulps [3–5]. Regenerative endodontics concept was initially introduced by Östby1961 [6]. Since then, various protocols for regenerative endodontic procedures were proposed [7, 8], differing mainly in disinfection strategies, intracanal medicaments, scaffold systems, and restorative materials [9–11]. To minimize such variability, evidence-based clinical guidelines have been issued by the American Association of Endodontists (AAE) and the European Society of Endodontology [3, 4]. Nevertheless, differences in treatment protocols and outcome assessment methods persist due to the limited availability of high-quality clinical evidence.

The favorable outcomes of regenerative endodontic techniques in immature necrotic teeth, including resolution of clinical manifestations, healing of the periapical tissues, and continuation of root maturation, have encouraged extending these approaches in mature teeth with closed apices. However, most of the scientific evidence on pulp regeneration in mature teeth was coming from low evidence case reports or case series [12].

Among clinicians, blood clot–mediated revascularization remains the most commonly employed approach. Clinical studies have demonstrated that blood clots achieve success rates comparable to alternative scaffold materials [13]. However, blood clot formation may be unpredictable and provides a relatively limited source of growth factors, which may restrict its clinical applicability [14].

The regenerative protocol was subsequently enhanced with the introduction of first-generation platelet concentrates, such as platelet-rich plasma (PRP). Subsequently platelet-rich fibrin (PRF), a second-generation autologous platelet concentrates developed by Choukroun et al. [15].

In 2014, injectable platelet-rich fibrin (i-PRF) was introduced aimed to improve the biological properties of PRF [16]. This flowable form of PRF was produced by using plastic tubes and reduced centrifugation speeds for shorter periods of time (700 rpm for 3 min) [17]. After application, fibrinogen rich fluid in i-PRF gradually converts into a growth factor–rich PRF clot, which continuously liberate these factors over a period of 10–14 days, comparable to the PRF which provide sustained growth factors release up to 7 days after its application [18].

In addition to platelets, i-PRF contains leukocytes, chemotactic cytokines that can support dental pulp regeneration [19, 20]. i-PRF has also demonstrated enhanced anti-inflammatory and antimicrobial properties against various pathogens, which may further accelerate tissue healing [21].

Due to its liquid form, i-PRF can be used alone or combined with bioactive molecules such as vitamin C. Vitamin C, also referred to as L-ascorbic acid, is a water-soluble micronutrient essential for collagen synthesis and extracellular matrix formation [22]. Experimental studies have demonstrated that vitamin C enhances the proliferative capacity of mesenchymal stem cells and promotes collagen synthesis at concentrations up to 200 µM [23, 24]. These findings highlighting its potential role in regenerative dental therapies.

Despite the promising biological characteristics of injectable platelet-rich fibrin, the potential synergistic effect of combining i-PRF with vitamin C in regenerative endodontic therapy has not been sufficiently evaluated, particularly in mature necrotic teeth with closed apices. Moreover, evidence comparing CBCT with conventional radiographs in regenerative endodontic outcomes remains limited [25].

Accordingly, the primary objective of the present trial was to assess the healing potential of injectable platelet-rich fibrin combined with vitamin C as a scaffold in regenerative endodontic procedure of mature necrotic single-rooted teeth using CBCT. The secondary aims were to compare CBCT radiographic outcomes with those obtained from two-dimensional periapical radiographs, as well as to assess postoperative pain and clinical outcomes following regenerative endodontic procedures. The null hypothesis stated that no significant differences would be observed in radiographic healing, clinical outcomes, or postoperative pain between the two groups.

## Materials and methods

### Study design and registration

This randomized controlled trial was reported in accordance with the Consolidated Standards of Reporting Trials CONSORT 2025 guidelines (http://www.consort-statement.org/).

The study was conducted as a prospective, parallel-group, double-blinded, randomized controlled trial with a 1:1 allocation ratio. The trial was registered at ClinicalTrials.gov with (ID: NCT05585359) Registered on 14 October 2022, available at https://clinicaltrials.gov/study/NCT05585359, following the approval by the institutional review board of the faculty of Dentistry, Cairo University (approval number 4-1-23). Written informed consent was obtained from all participants prior to their enrollment in the study. Participant recruitment, treatment, and follow-up were conducted at outpatient clinic during the period between, April 2023 and January 2025. The patients were treated according to declaration of Helsinki as revised in 2013. No modifications to the study methodology, including eligibility criteria or outcome measures, were made after trial commencement.

### Sample size calculation

Sample size estimation was determined according to the primary outcome measure using data from a previous study [26]. According to that study, at least 12 teeth (12 participants) were required per group, assuming a normally distributed outcome, with a standard deviation of 5 and an expected mean difference of 6.

The calculation was performed to attain 80% statistical power with a significance level (α) of 0.05. To compensate for an anticipated dropout rate of 15%, the sample size increased to 14 teeth (14 participants) per group, resulting in a total sample of 28 teeth (28 participants). No interim analyses were performed, and no predefined stopping guidelines were established for this trial.

### Randomization and allocation concealment

Subjects were randomly allocated to either the study group, in which regenerative endodontic procedures were conducted using (i-PRF + AA), or (i-PRF) alone was used.

A computer-generated simple random allocation sequence (1–28) was generated and organized into a two-column table (A and B) (http://www.random.org/), representing the experimental and control groups, respectively.

The allocation sequence was generated randomly and maintained by an independent investigator who had no involvement in patient recruitment, treatment, or outcome assessment. Allocation concealment was ensured using sequentially numbered, sealed opaque envelopes, which were opened only at the time of intervention. The personnel responsible for participant enrollment and treatment assignment had no access to the random allocation sequence before allocation.

### Blinding

Operator blinding was not feasible due to the nature of the interventions. However, participants were masked to the assigned intervention or control group, as the preparation of injectable platelet-rich fibrin, with or without the addition of Vitamin C supplementation, was performed out of their view following blood withdrawal.

Radiographic outcome evaluation was carried out by two independent blinded examiners who were not involved in the treatment procedures and were unaware of group allocation. In addition, the statistician was blinded to group allocation during data analysis.

### Eligibility criteria

Patients were eligible if they were medically healthy (ASA physical status I or II), aged between 18 and 45 years, with no sex predilection, and presented with mature permanent maxillary or mandibular anterior teeth diagnosed with pulp necrosis. Eligible teeth exhibited a negative response to cold testing and were accompanied by swelling or without swelling and/or a sinus tract, along with radiographic evidence of periapical radiolucency. All included teeth had fully developed roots with closed apices.

Exclusion criteria included patients who were medically compromised (ASA physical status III or IV) or those with a history of allergy to any of the materials or medications used in the study. Teeth were excluded if they presented with periodontal pockets, pathological mobility, non-restorable crowns, or required post space for restoration. Additionally, teeth presenting with a history of previous root canal treatment, developmental abnormalities, or internal and external root resorptive defects were excluded.

### Setting and operator details

The trial was conducted at university-based outpatient clinic, Faculty of Dentistry, Cairo University, Egypt. All clinical procedures and data collection were carried out in the same clinical setting under standardized conditions. All interventions were accomplished by the same calibrated operator, an experienced endodontist with a Master’s degree in Endodontics, following a standardized treatment protocol to ensure consistency and minimize operator-related variability.

### Diagnosis

**Fig. 1:**
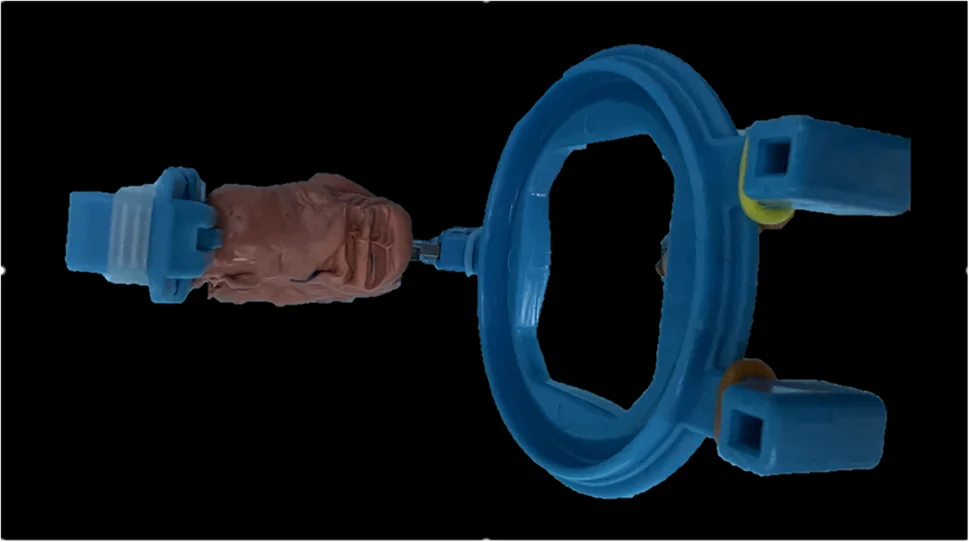
Custom-made index comprising a Rinn film holder, a paralleling device and silicone impressions

All procedures were conducted by the single operator and were standardized across both groups to ensure reproducibility and comparability as follows: pulpal necrosis in single-rooted teeth associated with periapical radiolucency was clinically and radiographically verified. A preoperative periapical radiograph was obtained using the standardized paralleling technique to confirm the presence of periapical radiolucency (Fig. 1). Subsequently, cone-beam computed tomography (CBCT) was performed preoperatively to measure the size of the periapical lesion (mm²) and to assess its extent and boundaries in three dimensions, including the buccopalatal, mesiodistal, and diagonal planes.

#### First visit

Local anesthesia was achieved using 4% Articaine with 1:200,000 epinephrine (Septanest; Septodont, Saint-Maur-des-Fossés, France). Following rubber dam isolation (Sanctuary Health SDN BHD, Chemor, Perak, Malaysia) and access cavity preparation, working length was established using an electronic apex locator (Root ZX-II; J. Morita MFG Corporation, Fushimi-ku, Kyoto, Japan) and verified radiographically (Nanopix, Eighteeth, China).

Root canal preparation was carried out using a crown-down technique with MG3 nickel-titanium rotary files (Meta Biomed, Cheongju, Korea). The final apical preparation size was standardized to 35/0.04 for mandibular anterior teeth and 40/0.04 for maxillary anterior teeth, and increased to 50/0.04 when the initial apical binding file exceeded file size 25. All instrumentation was conducted using an endodontic motor in accordance with the manufacturer’s recommendations (Denjoy i-mate3, Denjoy Dental CO., China).

The root canals were irrigated between successive files with 1.5% sodium hypochlorite (NaOCl) 20 mL over 5 min using a 30-gauge single side-vented needle positioned approximately 3 mm short of the root apex. Irrigant was activated in three consecutive cycles of 20 s each (Ultra X device), the canals were subsequently flushed with 5 mL sterile saline and dried with paper points.

Double antibiotic paste (DAP) was prepared by pulverizing one 500-mg tablet of metronidazole and one 500-mg tablet of ciprofloxacin in a 1:1 ratio and mixing them with sterile saline to obtain homogeneous paste with a cream-like consistency. The paste was delivered into the canal using a hypodermic needle and positioned slightly apical to the cemento–enamel junction.

A sterile dry cotton pellet was placed over the paste, followed by restoration of the tooth with modified glass ionomer filling (SDI company, Germany) for 3 weeks.

#### Second visit and randomization

The patients were anesthetized with same anesthesia type as the first appointment, subsequently the tooth was isolated and the intermittent restoration was removed. The double antibiotic paste was eliminated using copious irrigation with 5 mL of sterile saline.

The final irrigation protocol consisted of 1.5% sodium hypochlorite (NaOCl) 20 mL over 5 min, with activation (three cycles of 20 s each), followed by 10 mL of 17% EDTA solution (Cerkamed Dental Products, Stalowa Wola, Poland). The solutions were separated by 20 mL of sterile saline irrigation. The canals were then dried using sterile paper points.

Participants were then randomly allocated into two groups. The subsequent procedures were carried out according to group assignment, as follows:

#### Experimental group: injectable platelet-rich fibrin combined with vitamin C (i-PRF + AA)

Upon removal of the double antibiotic paste and drying of the canal, 10 mL of whole venous blood were collected from the patient’s median cubital vein and transferred into plain plastic test tubes that didn’t contain anticoagulant.

The samples were immediately centrifuged after collection to prevent premature coagulation at 700 rpm for 3 min (VE-4000, USA) at room temperature [17].

The upper yellow layer, identified injectable platelet-rich fibrin (i-PRF), was carefully collected using a sterile 30 G syringe, positioned as close as possible to the red cell layer.

Each ampoule of vitamin C contained 500 mg/5 mL, corresponding to 100 mg/mL, which is approximately 567 µM per 1 mL; thus, 0.5 mL contains roughly 250 µM of Vitamin C.

Based on the volume of injectable platelet-rich fibrin (i-PRF) collected following centrifugation, the corresponding amount of vitamin C was calculated. For each 1 mL of i-PRF, 0.5 mL (≈ 250 µM) of vitamin C (C-Loges, Germany) was mixed with the i-PRF [27].

The mixture was then injected into the canals using a plastic syringe fitted with a 30-gauge side-vented needle, which was inserted 3 mm short of the working length and gradually withdrawn while injecting, until the canal orifice was reached. The mixture (i-PRF + AA) was retained in the canal for 15 min to permit partial hardening before the application of the bioactive and restorative materials.

#### Control group: injectable platelet-rich fibrin alone (i-PRF)

The same protocol was followed as in the experimental group. Following blood withdrawal, 10 mL of venous blood was obtained and immediately centrifuged at 700 rpm for 3 min at room temperature [17] under identical conditions.

The i-PRF layer was collected using the same technique and delivered into the canal in the same manner. The material was retained in the canal for 15 min to permit partial harden before placement of the coronal barrier.

The timing of intervention, method of preparation, and mode of application were identical to those of the experimental group. The only difference between groups was the absence of Vitamin C in the control group.

### Placement of bioactive and restorative materials

After completion of the regenerative procedure in both groups, a collagen plug was placed over the scaffold to limit apical extrusion of the bioactive material. A 3-mm layer of premixed calcium silicate bioceramic material (NeoPUTTY, Avelon Biomed, USA) was applied at the cemento-dentinal junction, this was followed by reinforced glass ionomer (RIVA Self-Cure, SDI Limited, Bayswater, Victoria, Australia) and sealed with resin composite (Ivoclar Vivadent, Liechtenstein) to complete the restoration of the access cavity.

### Outcome assessment

The outcome assessment was based on the established framework by the American Association of Endodontists (AAE) [3], according to which the outcomes of regenerative endodontic procedures can be considered in terms of primary, secondary, and tertiary goals. Resolution of symptoms and periapical healing represent the primary goals, whereas increased root wall thickness and/or root length are considered secondary goals, which is desirable but not essential and a positive response to sensibility testing represents a tertiary goal, which if achieved, could indicate the presence of vital intracanal tissue.

In the present study, the primary outcome measure was radiographic healing of the periapical lesion, assessed quantitatively by measuring the lesion area (mm²) using CBCT at baseline and 12 months. Secondary outcomes included radiographic healing assessed using CBCT periapical index scores (CBCT-PAI), two-dimensional radiographic assessment of lesion area, clinical outcomes, and postoperative pain.

Clinical outcomes included pain on percussion, swelling, and the presence of a sinus tract. Clinical success was defined as the absence of all clinical signs and symptoms. Clinical success was considered an additional clinical outcome and was interpreted alongside the radiographic assessment of periapical healing rather than as a standalone indicator of regenerative success.

For postoperative pain assessment, patients were provided with a standardized pain chart (Numerical Pain Rating Scale) to record their pain levels at the specified time intervals. In addition, patients were contacted by phone at each scheduled time point to remind them, and ensure accurate reporting of their pain scores, thereby minimizing recall bias and improving compliance.

Radiographic measurements for both CBCT and two-dimensional radiographs were assessed by two independent, calibrated assessors, and the mean of their measurements was used as the final recorded value. Clinical examinations and postoperative pain assessments were carried out by a calibrated investigator.

### CBCT assessment

**Fig. 2:**
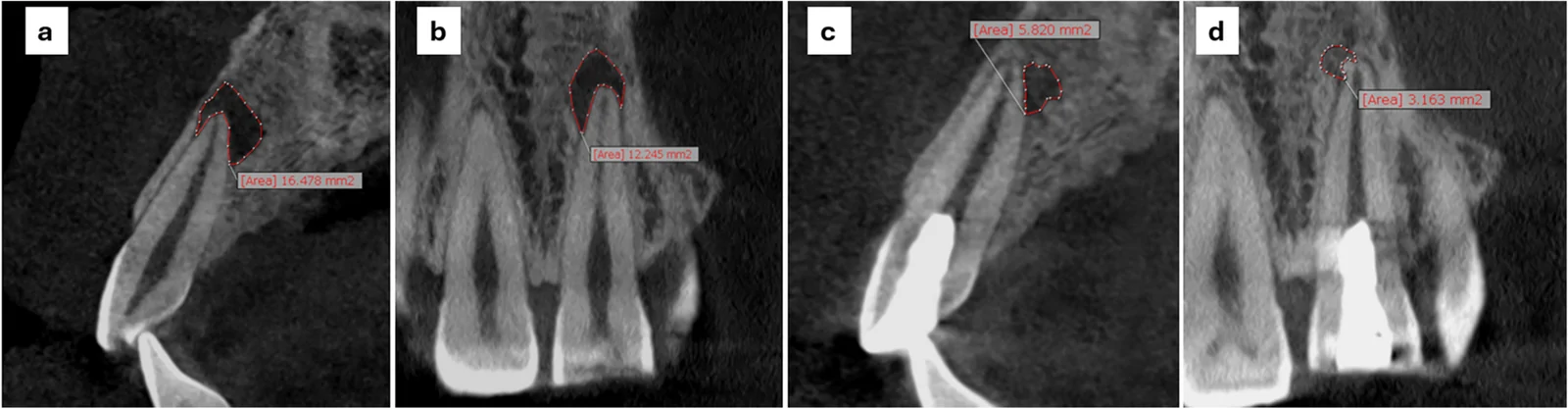
Preoperative and postoperative cone-beam computed tomography (CBCT) images demonstrating periapical lesion area measurements (mm²) using the area measurement tool of the OnDemand CBCT software: (**a**) preoperative sagittal view, (**b**) preoperative coronal view, (**c**) postoperative sagittal view and (**d**) postoperative coronal view. The outlined region represents the boundaries of the periapical radiolucent lesion used for area measurement. A progressive reduction in lesion size is observed over time, indicating favorable periapical healing

CBCT scans were obtained at baseline and 12 months postoperatively using the same CBCT unit (Morita, Japan) with standardized parameters (90 kVp, 8 mA, 6.1-s exposure time, 125-µm voxel size, 5 × 5 cm field of view, and 1.5-mm slice thickness). Bone healing was evaluated by measuring the periapical lesion area (mm²) in both sagittal and coronal planes using the area measurement tool of the OnDemand CBCT software (Fig. 2a-d). The final lesion area was calculated as the mean of the two measurements.

The use of CBCT imaging was justified to ensure accurate three-dimensional assessment of periapical healing and lesion size while adhering to the ALARA principle.

**Fig. 3:**
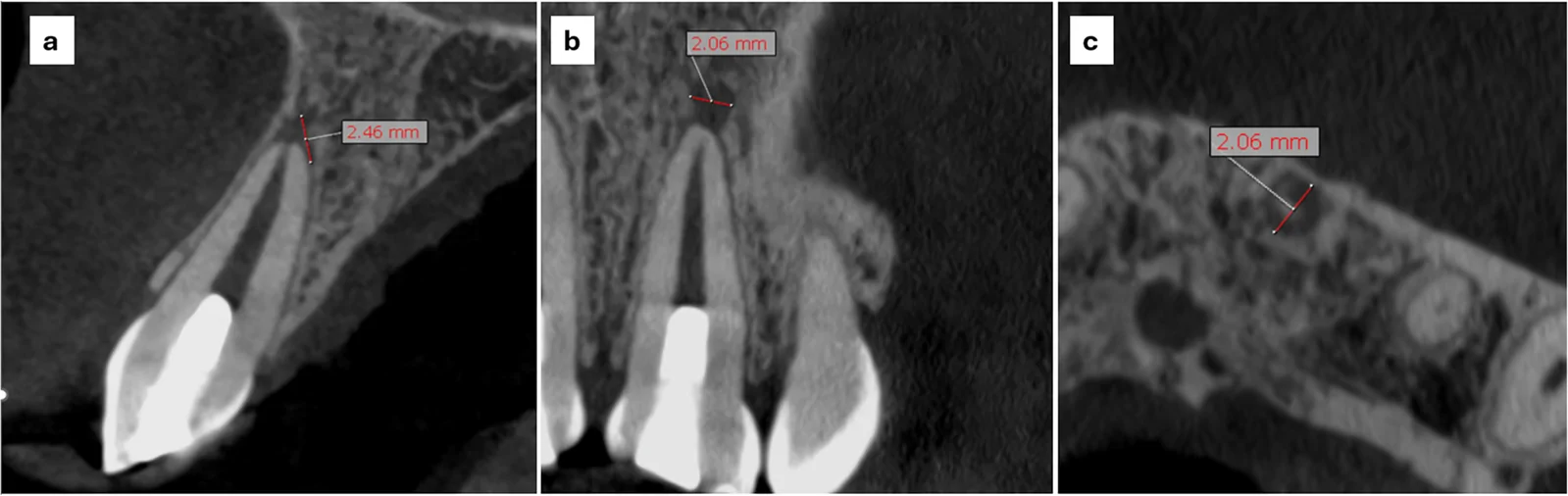
Postoperative cone-beam computed tomography (CBCT) images demonstrating periapical lesion linear measurements (mm) in three planes for determination of the CBCT-PAI score: (**a**) Postoperative sagittal view, (**b**) postoperative coronal view and (**c**) postoperative axial view. The linear measurements shown represent the maximum diameter of the lesion in each plane

Beside the previously described outcome measure, periapical healing was assessed according to the method described by Estrela et al. [28]. Linear measurements (mm) of the periapical lesion were obtained at baseline and at 12 months in three dimensions-axial, coronal, and sagittal planes using the OnDemand CBCT software (Fig. 3a-c). After the lesion was measured, the final score was based on the greatest lesion extension and subsequently categorized as follows:

- 0 = Intact periapical bone structure.
- 1 = Diameter of periapical radiolucency > 0.5–1 mm.
- 2 = Diameter of periapical radiolucency > 1–2 mm.
- 3 = Diameter of periapical radiolucency > 2–4 mm.
- 4 = Diameter of periapical radiolucency > 4–8 mm.
- 5 = Diameter of periapical radiolucency > 8 mm.
- Score (*n*) + E = Expansion of the cortical bone.
- Score (*n*) + D = Destruction of the cortical bone.

### Two-dimensional radiographic assessment

Two-dimensional radiographic evaluation was carried out using standardized digital periapical radiographs obtained at baseline and every 3 months for 1 year (Nanopix, Eighteeth, China). Standardization was achieved using a tailor-made positioning index consisting of a Rinn film holder and a paralleling device, together with silicone impression material (Fig. 1).

**Fig. 4:**
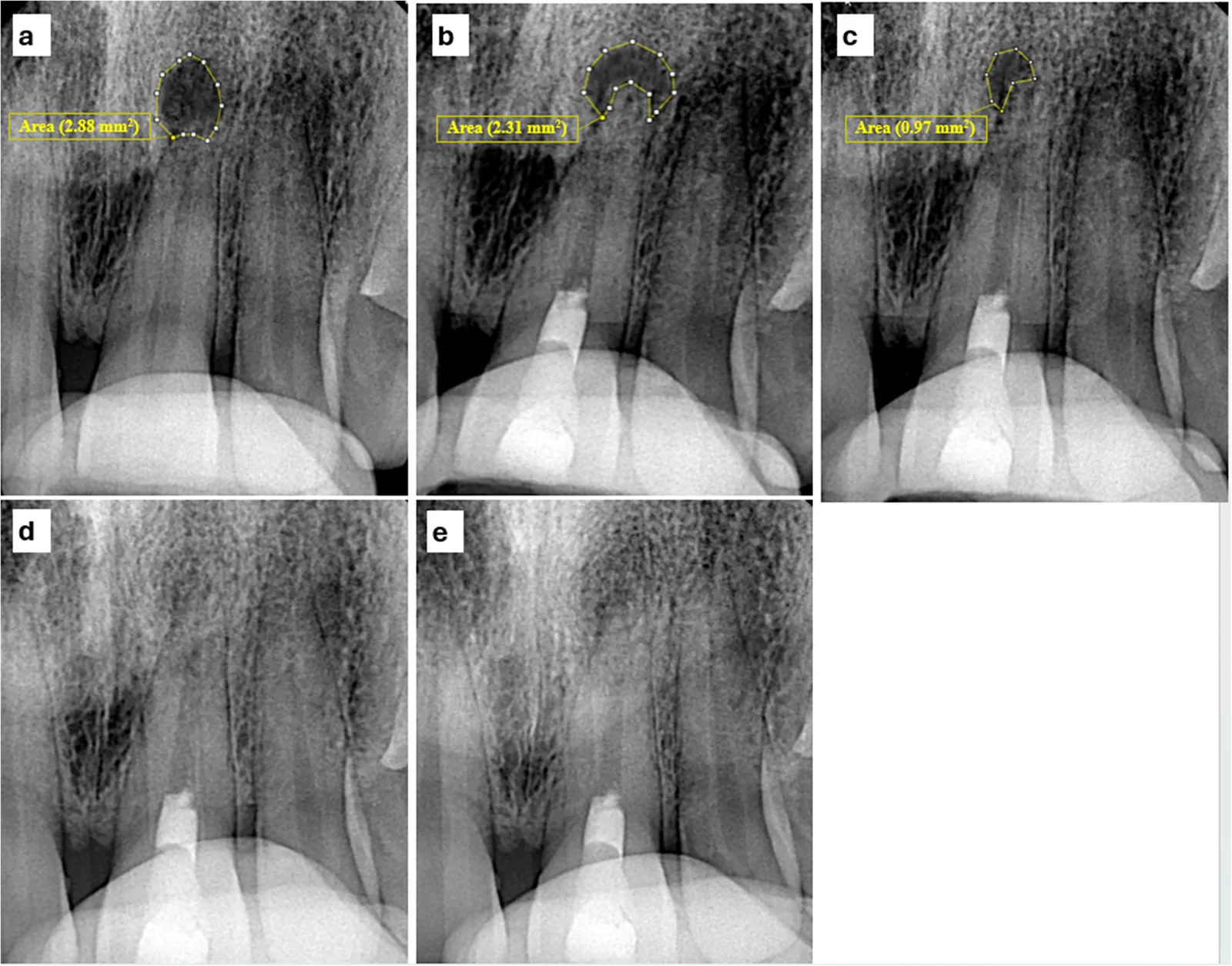
Standardized two-dimensional periapical radiographs obtained using a paralleling device and rubber silicone bite block at different time intervals, demonstrating periapical lesion area measurements (mm²) using the polygonal selection tool in ImageJ software: (**a**) Pre-treatment (baseline) periapical radiograph, (**b**) 3-month post-treatment, (**c**) 6-month post-treatment, (**d**) 9-month post-treatment and (**e**) 12-month post-treatment. The outlined region represents the boundaries of the periapical radiolucent lesion used for area measurement. A progressive reduction in lesion size is observed over time, indicating favorable periapical healing

The area of the periapical lesion (mm^2^) was measured using the polygonal selection tool in ImageJ software (version 1.50i; National Institutes of Health, Bethesda, MD, USA) (Fig. 4a-e), as previously described by Bose et al. and Flake et al. [29, 30]. Radiographic images were evaluated under standardized conditions using a high-resolution monitor. Measurements were performed independently. No image enhancement was applied. Patient’s names, ages were removed from radiographic images prior to analysis ensure confidentiality.

### Clinical assessment

Clinical examinations were performed throughout the follow-up period at 3, 6, 9, and 12 months. The following parameters were assessed: Tenderness to percussion, swelling, presence or absence of sinus tract. Clinical success was defined as the absence of all the previous parameters.

### Postoperative pain assessment

Patients were instructed to mark the number that best represented their perceived pain intensity using the “Numerical Pain Rating Scale” at 6, 12, 24, 48, and 72 h, and at 1 week following the second visit. The NRS is a 10-point scale ranging from 0 to 10 and was categorized into four levels as described by Williamson and Hoggart [31]: no pain (0), mild pain (1–3), moderate pain (4–6), and severe pain (7–10).

No modifications to the predefined primary or secondary outcomes were made after the commencement of the trial.

### Adverse events assessment

Adverse events and harms were systematically assessed throughout the follow-up period by clinical examination and patient-reported symptoms, including postoperative pain, swelling, tenderness to percussion, and sinus tract formation.

### Statistical analysis

Numerical data were assessed for normality by examining their distribution and using the Kolmogorov-Smirnov and Shapiro-Wilk tests. All variables demonstrated a non-normal (non-parametric) distribution. Accordingly, numerical data were presented as median, range, mean, and standard deviation (SD).

Comparisons between the two groups were performed using the Mann-Whitney U test. Changes within each group were analyzed using the Wilcoxon signed-rank test and Friedman’s test. When Friedman’s test indicated significance, Dunn’s test was applied for pairwise comparisons.

Categorical data were expressed as frequencies and percentages, and comparisons between groups were conducted using Fisher’s exact test.

The level of consensus between raters for the CBCT and two-dimensional radiographic methods was evaluated using Cronbach’s alpha reliability coefficient and the intra-Class Correlation Coefficient (ICC). Cronbach’s Alpha values were interpreted as follows: 0–0.2, weak agreement; 0.2–0.4, fair agreement; 0.4–0.6, moderate agreement; 0.6–0.8, good agreement; 0.8–0.99, very good agreement; and 1, perfect agreement.

Statistical significance was set at *P* ≤ 0.05. All analyses were conducted using IBM SPSS Statistics for Windows, Version 23.0 (Armonk, NY: IBM Corp).

The analysis was performed on participants who completed the study and were analyzed according to their assigned intervention groups. Missing data due to drop-outs were excluded from the analysis, and no imputation methods were applied. No subgroup or adjusted analyses were performed in this study. This study did not involve clustered data; therefore, no adjustment for cluster effects was necessary.

## Results

A total of 28 participants were randomly allocated into two groups (*n* = 14 per group). Baseline demographic and clinical characteristics were available for all 28 randomized participants. During follow-up, two participants from the control group were not available for the periapical healing assessment. One participant could not be contacted at the 6-month follow-up despite attempts to reach the participant. The second participant reported fracture of the treated tooth before the scheduled 3-month follow-up. Clinical examination revealed fracture of the restoration with loss of part of the tooth structure and the presence of a sinus tract. The case was considered as a treatment failure due to reinfection and was subsequently managed with conventional root canal treatment and referred to the prosthodontic department for definitive restoration.

**Fig. 5:**
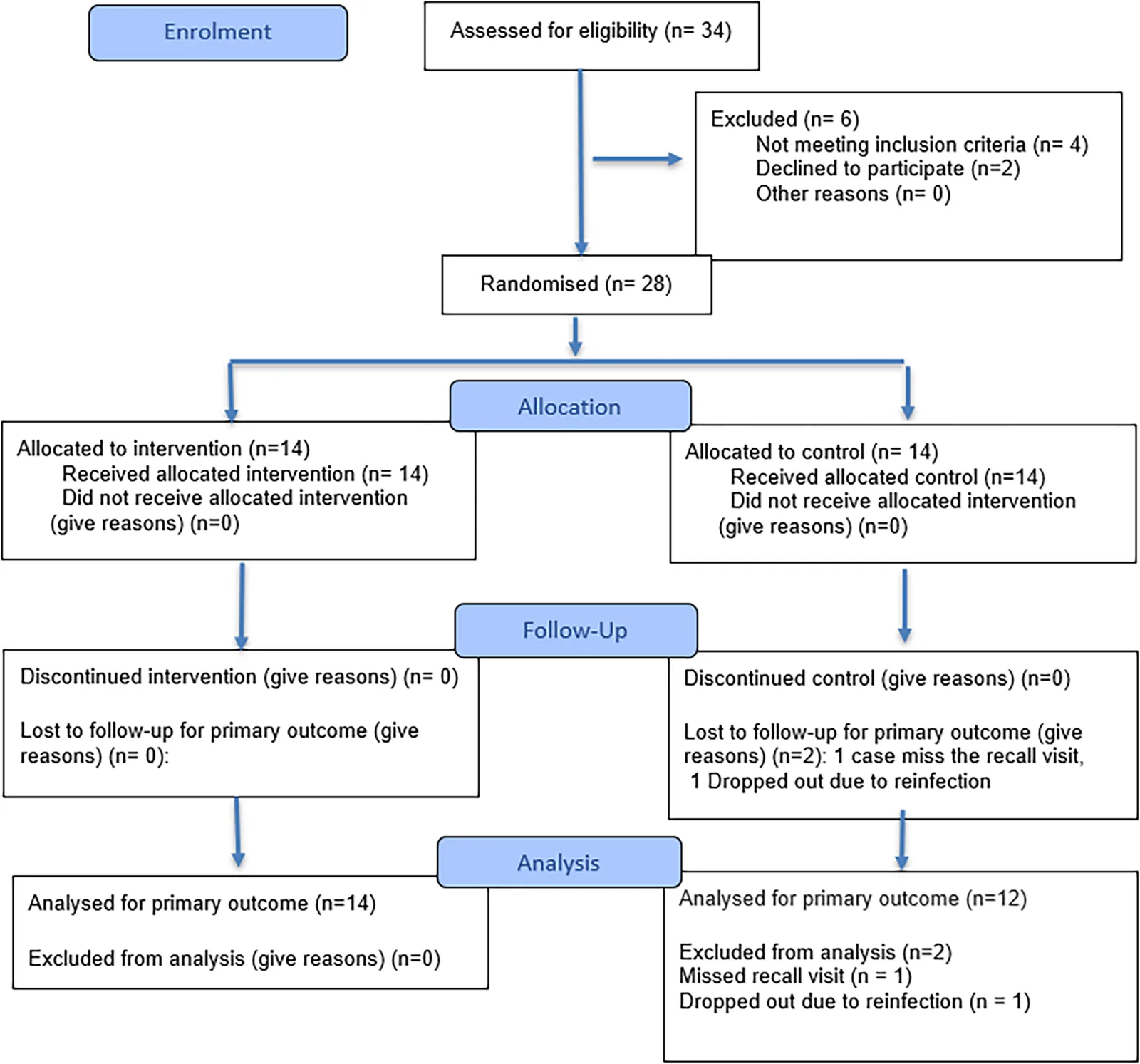
CONSORT 2025 flow diagram

Accordingly, periapical healing and clinical normalcy were evaluated in 14 participants in the intervention group and 12 participants in the control group. In contrast, baseline characteristics and postoperative pain data were available for all 28 participants (14 participants per group). All outcome analyses were performed using a complete-case approach. A flow diagram illustrating participant recruitment, allocation, follow-up, and analysis is presented in (Fig. 5).

Patient recruitment was conducted from April 2023 to January 2025, and the follow-up period extended to 12 months. The study was completed as planned, with no early termination.

Both interventions were delivered as planned according to the standardized treatment protocol, with no deviations from the assigned interventions. No additional concomitant treatments were provided during the trial, and all participants received the same standard care protocol.

### Baseline characteristics

The baseline characteristics of participants in both study groups are presented in (Table 1). Baseline characteristics were assessed prior to drop-outs. No statistically significant differences were observed between groups regarding age, gender and tooth type (*P* > 0.05).

**Table 1:** Baseline characteristics of the study groups

Base line characteristics	Intervention (*n* = 14)	Control (*n* = 14)
Gender [*n*, (%)]		
Male	5 (35.7%)	5 (35.7%)
Female	9 (64.3%)	9 (64.3%)
Age [Mean, SD]	33.2 (6.1)	35.3 (7.8)
Tooth [*n*, (%)]		
Central incisor	11 (78.6%)	9 (64.3%)
Lateral incisor	3 (21.4%)	5 (35.7%)

Values are presented as number (percentage) or mean (SD)

*SD* Standard deviation

### Inter-observer agreement

For the CBCT lesion area measurements (mm²), both groups showed near-perfect reliability at pre-operative and post-operative assessments, with Cronbach’s alpha values ranging from 0.999 to 1.000 and intraclass correlation coefficients (ICC) ranging from 0.998 to 0.999. Regarding the CBCT-PAI scores, very good inter-observer agreement was observed in both groups, with Cronbach’s alpha values ranging from 0.983 to 0.999. For the two-dimensional radiographic assessment, the intervention group demonstrated moderate to very good agreement (Cronbach’s alpha: 0.589–0.999), while the control group showed very good to perfect agreement (Cronbach’s alpha: 0.985–1.000). Overall, these findings indicate high reliability and consistency between observers across all evaluation methods (Table 2).

**Table 2:** Inter-observer agreement across different imaging modalities and time points

Outcome	Time	Intervention (*n* = 14) Cronbach’s α	ICC	Control (*n* = 12) Cronbach’s α	ICC
CBCT lesion area (mm²)	Pre-op Post-op	1.000 0.999	0.999 0.998	0.999 0.999	0.998 0.999
CBCT-PAI Scores	Pre-op Post-op	0.991 0.997	0.983 0.994	0.983 0.999	0.966 0.998
2D radiographic lesion area (mm²)	Pre-op 3 months	0.997 0.998	0.995 0.997	0.992 1.000	0.984 0.999
	6 months	0.996	0.993	1.000	1.000
	9 months	0.999	0.998	0.985	0.971
	12 months	0.589	0.417	0.999	0.998

*ICC* Intraclass Correlation Coefficient

### CBCT outcome (lesion area in mm^2^)

#### Comparison between the two groups

At baseline, no statistically significant difference was observed between the intervention and control groups. The median pre-operative values were 28.7 (range: 6.7–64.6) in the intervention group and 29.0 (range: 1.3–80.0) in the control group, with corresponding mean values of 27.6 ± 17.7 and 32.2 ± 23.1, respectively, with no statistically significant difference (*P* = 0.734), and small effect size (Cohen’s d = 0.245), indicating comparable baseline between the groups.

Following treatment, the post-operative median values were 3.5 (range: 0–26.8) for the intervention group and 5.3 (range: 0–27.9) for the control group, with corresponding mean values of 8.3 ± 9.5 and 7.4 ± 8.2, respectively, no statistically significant difference (*P* = 0.981), and negligible effect size (d = 0.131), as shown in (Table 3).

**Table 3:** Comparison of lesion area measurements (mm^2^) between the intervention and control groups and within-group changes over time

Time	Intervention (*n* = 14)		Control (*n* = 12)		*P*-value	Effect size (d)
	Median (Range)	Mean (SD)	Median (Range)	Mean (SD)		
Pre-operative	28.7 (6.7, 64.6)	27.6 (17.7)	29 (1.3, 80)	32.2 (23.1)	0.734	0.245
Post-operative	3.5 (0, 26.8)	8.3 (9.5)	5.3 (0, 27.9)	7.4 (8.2)	0.981	0.131
*P*-value	0.001*		0.001*			
*Effect size (d)*	0.881		0.85			

*Statistically significant at *P* < 0.05

#### Changes within each group

However, within-group analysis demonstrated a statistically significant reduction from pre-operative to post-operative values in both groups (*P* = 0.001 for each) (Table 3). The magnitude of change was large in the intervention group (d = 0.881) and in the control group (d = 0.85), indicating a substantial improvement over time irrespective of the treatment modality.

#### Comparison between amounts of reduction in lesion area in the two groups

**Fig. 6:**
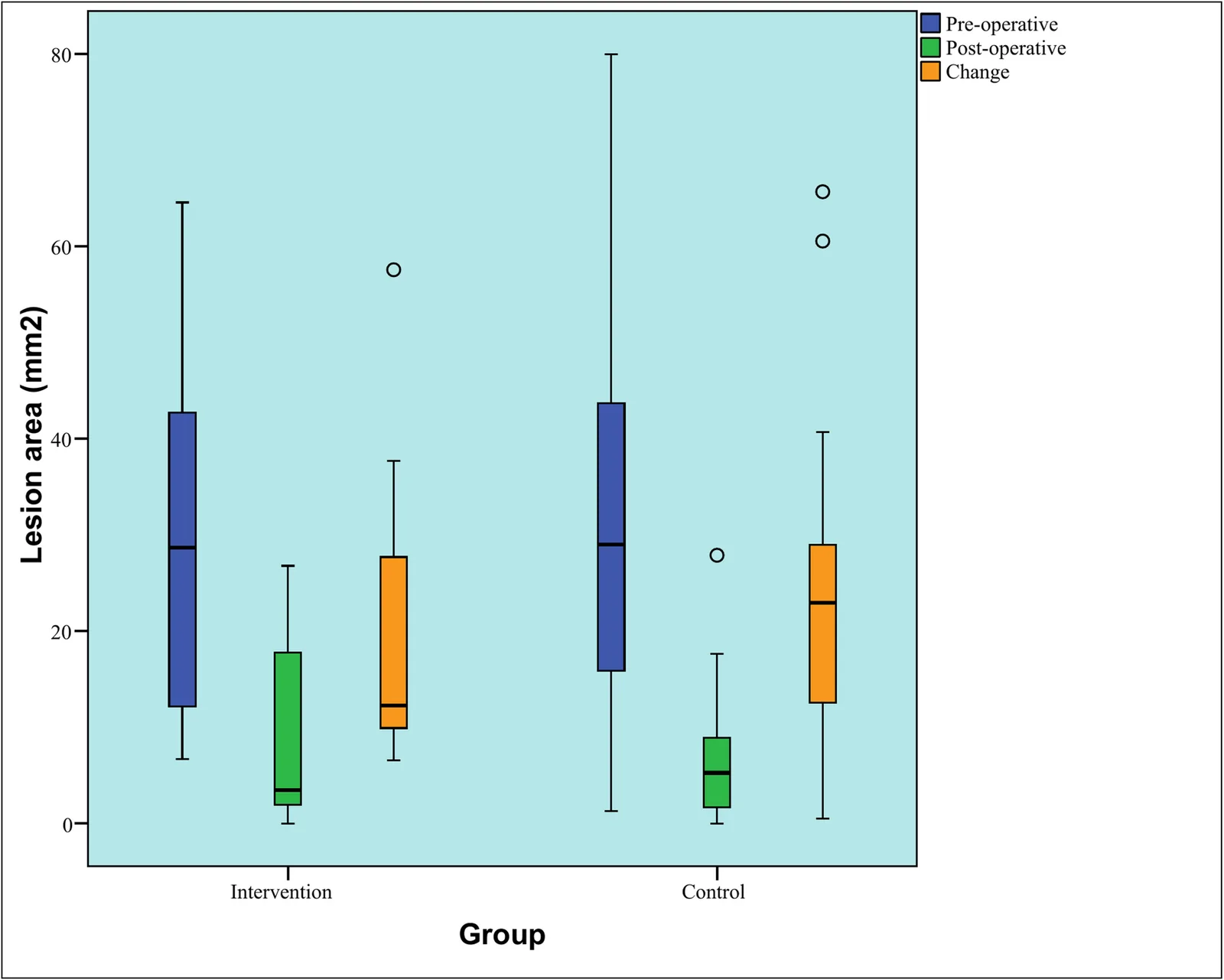
Box plot illustrating the distribution of lesion area measurements in CBCT (mm²) in the intervention and control groups, (circles represent outliers)

The intervention group demonstrated a lower median value of (12.3) compared to control group showed a higher median value of (22.9). However, there was no statistically significant difference in the amount of lesion area reduction between the two groups (Table 4), (*P*-value = 0.497, Effect size = 0.371) (Fig. 6).

**Table 4:** comparison between amounts of reduction in lesion area (mm^2^) in the two groups

Intervention (*n* = 14)		Control (*n* = 12)		*P*-value	Effect size (d)
Median (Range)	Mean (SD)	Median (Range)	Mean (SD)		
12.3 (6.6, 57.6)	19.3 (14.9)	22.9 (0.5, 65.7)	24.8 (20.1)	0.497	0.371

Statistically significant at *P* < 0.05

### CBCT-PAI outcome

#### Comparison between the two groups

At the preoperative assessment, there was no statistically significant difference in the index scores between the intervention and control groups (*P* = 0.767), with a moderate effect size (Cramér’s V = 0.449) as shown in (Table 5).

**Table 5:** Comparison of PAI scores between the intervention and control groups, and within-group changes

Time	Score	Intervention (*n* = 14)		Control (*n* = 12)		*P*-value	Effect size (v)
		*n*	%	*n*	%		
Pre-operative	3	1	7.1	1	7.7	0.767	0.449
	3 + D	1	7.1	7	7.7		
	4	2	14.3	0	0		
	4 + D	3	21.4	2	15.4		
	4 + E	1	7.1	0	0		
	5	1	7.1	4	30.8		
	5 + D	1	7.1	2	15.4		
	5 + E	4	28.6	3	23.1		
Post-operative	0	3	21.4	1	7.7	0.925	0.397
	2	1	7.1	1	7.7		
	2 + D	1	7.1	1	7.7		
	3	2	14.3	3	23.1		
	3 + D	1	7.1	0	0		
	4	6	42.9	5	38.5		
	4 + D	0	0	1	7.7		
	Failure	0	0	1	7.7		
*P*-value		0.181		0.319			
*Effect size (d)*		0.358		0.277			

Statistically significant at *P* < 0.05

Postoperatively, improvement in outcome scores was observed in both groups; however, no statistically significant difference was detected between the intervention and control groups (*P* = 0.925), with a moderate effect size (Cramér’s V = 0.397). The majority of cases in both groups achieved favorable postoperative scores.

#### Changes within each group

Within-group comparisons revealed no statistically significant differences between preoperative and postoperative scores for either the intervention group (*P* = 0.181; Cohen’s d = 0.358) or the control group (*P* = 0.319; Cohen’s d = 0.277), indicating small to moderate effect sizes (Table 5).

### Two-dimensional radiographic assessment

#### Comparison between the two groups

At the preoperative assessment, the intervention group demonstrated significantly smaller lesion size values compared with the control group (*P* = 0.007) with large effect size (Cohen’s d = 1.232). At 3, 6, 9, and 12 months, no statistically significant differences were observed between the 2 groups (*P* > 0.05). Nevertheless, the intervention group consistently demonstrated lower median and mean lesion area size values postoperatively, as shown in ( Table 6).

#### Change within groups

**Fig. 7:**
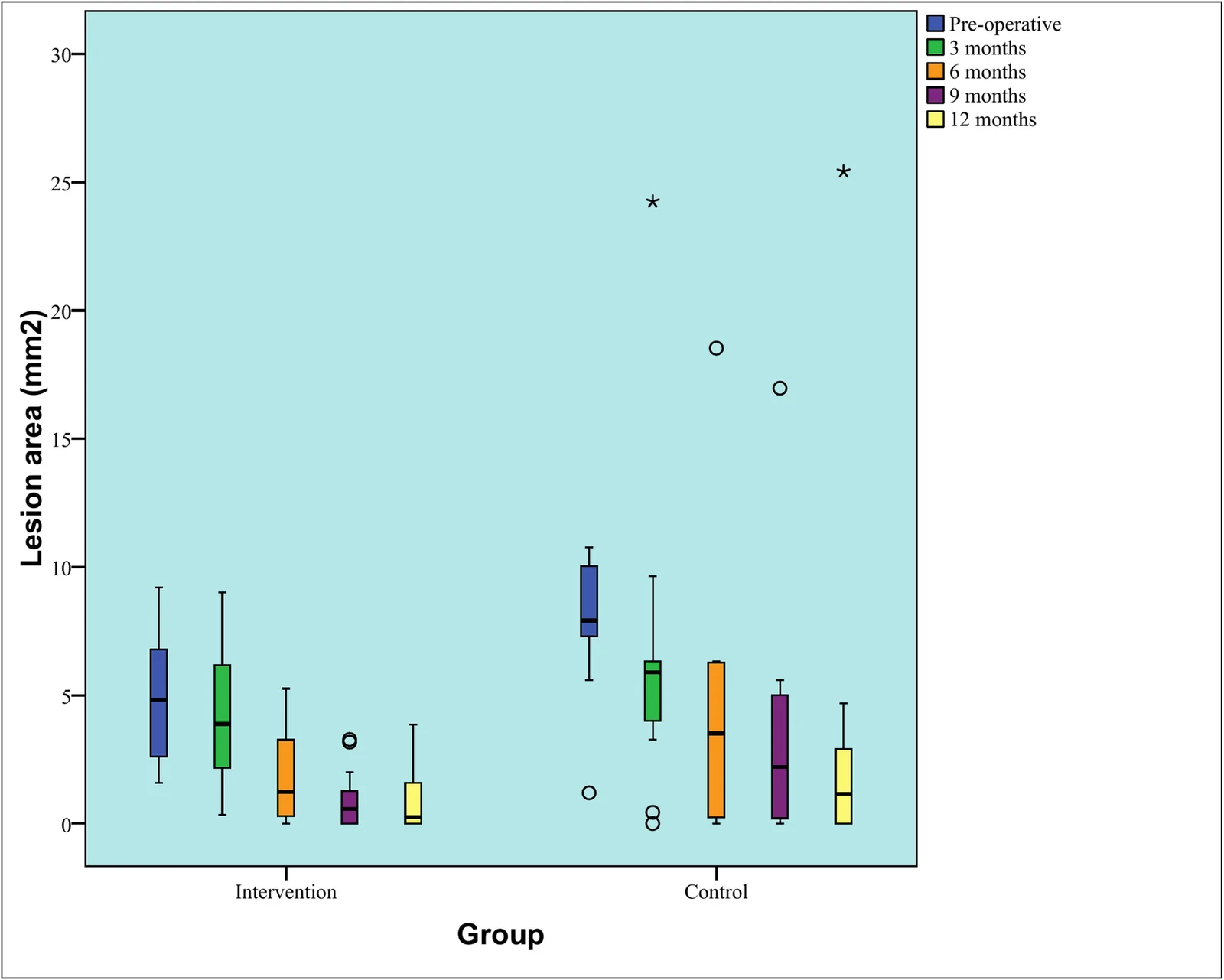
Box plot illustrating the distribution of lesion area measurements in two-dimensional radiograph (mm²) in the intervention and control groups, (circles represent outliers)

Both the intervention and control groups exhibited large and moderate to large effect size (w = 0.87 and w = 0.628 respectively), with a statistically significant reduction in lesion area over the follow-up period compared with baseline (*P* < 0.001). This was followed by non-significant change in lesion area from nine to 12 months. Indicating substantial lesion healing over time in both groups (Table 6). The temporal changes in lesion area measurements across different follow-up intervals in both groups are illustrated in (Fig. 7).

**Table 6:** comparison of 2D lesion area measurements (mm^2^) between the two groups and changes within each group

Time	Intervention (*n* = 14)		Control (*n* = 12)		*P*-value	Effect size (d)
	Median (Range)	Mean (SD)	Median (Range)	Mean (SD)		
Pre-operative	4.8 (1.6, 9.2)^A^	5 (2.5)	7.9 (1.2, 10.8)^A^	7.9 (2.6)	0.007*	1.232
3 months	3.9 (0.3, 9)^B^	4.1 (2.6)	5.9 (0, 24.3)^B^	6.5 (6.2)	0.217	0.499
6 months	1.2 (0, 5.3)^C^	1.9 (1.9)	3.5 (0, 18.5)^C^	4.3 (5.1)	0.235	0.477
9 months	0.6 (0, 3.3)^D^	0.9 (1.1)	2.2 (0, 17)^D^	3.6 (4.7)	0.055	0.805
12 months	0.3 (0, 3.9)^D^	0.9 (1.3)	1.2 (0, 25.4)^D^	3.4 (7.1)	0.239	0.455
*P*-value	< 0.001*		< 0.001*			
*Effect size (w)*	0.87		0.628			

*Significant at *P* ≤ 0.05, Different superscripts in the same column indicate statistically significant change within group

### Proportional lesion reduction

No statistically significant differences were observed between the intervention and control groups at any follow-up time point. At 3 months, the mean percentage change was − 10.9% in the intervention group compared with − 23.2% in the control group (*P* = 0.537). At 6 months, the corresponding values were − 64.3% and − 52.4%, respectively (*P* = 0.502). At 9 months, the intervention group showed a greater mean reduction than the control group (− 84.7% vs. −60.7%), although the difference did not reach statistical significance (*P* = 0.077). At 12 months, the mean percentage change was − 84.0% in the intervention group compared with − 64.4% in the control group (*P* = 0.556) (Table 7).

**Table 7:** (Proportion lesion reduction) comparison of percentage changes in 2D lesion area measurements (%) between the two groups

Time	Intervention (*n* = 14)		Control (*n* = 12)		*P*-value	Effect size (d)
	Median (Range)	Mean (SD)	Median (Range)	Mean (SD)		
3 months	-23.6 (-84.4, 184.9)	-10.9 (66.9)	-30.1 (-100, 138.5)	-23.2 (57.4)	0.537	0.244
6 months	-66 (-100, 41)	-64.3 (37.3)	-59.3 (-100, 82.2)	-52.4 (50)	0.502	0.265
9 months	-91.7 (-100, -51)	-84.7 (17.1)	-69.9 (-100, 66.9)	-60.7 (45.7)	0.077	0.73
12 months	-95.3 (-100, -35.7)	-84 (21.6)	-87.4 (-100, 150)	-64.4 (69.8)	0.556	0.223

Significant at *P* ≤ 0.05

The effect sizes were 0.244, 0.265, 0.73, and 0.223 at 3, 6, 9, and 12 months, respectively. The largest effect size was observed at 9 months (d = 0.73); however, the between-group difference remained statistically non-significant.

### Postoperative pain outcome

#### Comparison between the two groups

There was no statistically significant difference between the 2 groups at six as well as 12 h (*P*-value = 0.442, Effect size = 0.21) and (*P*-value = 0.150, Effect size = 0.245), respectively. Thereafter, no cases in either group reported a pain along the rest of follow up times (Table 8).

**Table 8:** Comparison between postoperative pain scores in the two groups and changes within each group

Time	Intervention (*n* = 14)		Control (*n* = 14)		*P*-value	Effect size (d)
	Median (Range)	Mean (SD)	Median (Range)	Mean (SD)		
6 h	0 (0, 7)^A^	0.9 (1.9)	0 (0, 5)	0.6 (1.5)	0.442	0.21
12 h	0 (0, 0)^B^	0 (0)	0 (0, 4)	0.2 (0.8)	0.150	0.245
24 h	0 (0, 0)^B^	0 (0)	0 (0, 0)	0 (0)	1	0
48 h	0 (0, 0)^B^	0 (0)	0 (0, 0)	0 (0)	1	0
72 h	0 (0, 0)^B^	0 (0)	0 (0, 0)	0 (0)	1	0
1 week	0 (0, 0)^B^	0 (0)	0 (0, 0)	0 (0)	1	0
*P*-value	0.001*		0.075			
*Effect size (w)*	0.286		0.143			

*Significant at *P* ≤ 0.05, Different superscripts in the same column indicate statistically significant change within group

#### Changes within each group

As regards intervention group, there was a statistically significant change in pain scores by time (*P* = 0.001; effect size, w = 0.286). Pair-wise comparisons between times revealed that there was a statistically significant decrease in pain scores after 6 h followed by non-statistically significant change from 12 h to one week. In contrast, no statistically significant change in postoperative pain over time-related changes were noted in the control group. (Table 8), (*P* = 0.075; effect size, w = 0.143).

### Clinical outcome

None of the cases in the two groups exhibited pain on percussion, swelling or sinus tract during the follow-up, therefore no statistical comparison was performed. Clinical success was achieved in 100% of cases in both groups (14/14 in the intervention group and 12/12 in the control group).

No subgroup or adjusted analyses were performed. No adverse or unintended events were observed in either group throughout the study period.

## Discussion

This randomized controlled trial demonstrates a good level of internal validity, achieved through a stringent study protocol, appropriate random allocation, standardized instrumentation and regenerative procedures, and the blinding of participants and outcome assessors, which further strengthens the reliability of the findings.

In terms of external validity, the regenerative scaffolds used, injectable platelet-rich fibrin either alone or combined with vitamin C, are clinically feasible and readily implementable in clinical practice. Moreover, the selected outcome measures such as periapical healing, clinical normalcy and postoperative discomfort are clinically relevant and patient-centered, suggesting that the findings are potentially generalizable to broader clinical populations, though replication in multicenter trials is warranted to confirm applicability.

Inclusion and exclusion criteria were carefully defined to ensure a homogeneous study population and to minimize potential confounding factors that could influence treatment outcomes. Patients included were mature permanent single rooted teeth with necrotic pulps and chronic apical periodontitis requiring regenerative endodontic procedures. Because certain physiological changes associated with aging, such as apical cementum thickening and apical foramen deviation may affect mesenchymal stem cell migration into the root canal system [32], the age range was limited to 18–45 years to minimize these effects. Both sexes were included without preferences, reflecting common clinical scenarios.

Exclusion criteria addressed conditions that could confound healing, including as systemic diseases or previous endodontic failures, resorptive defects and other different anomalies.

The follow-up schedule, incorporating early ( 3, 6 and 9 months) and longer-term assessments (12 months). The 12-month follow-up time was selected based on evidence indicating that periapical healing after regenerative endodontic procedures typically becomes radiographically and clinically apparent within 6–12 months [7, 33, 34].

Regenerative endodontic procedures (REPs) have recently been proposed as a treatment modality for mature permanent teeth [35, 36], aiming to overcome some of the limitations associated with conventional endodontic treatment [37, 38].

Several case reports have reported favorable clinical outcomes following REPs in mature teeth, including the resolution of signs and symptoms and successful healing of apical periodontitis [12, 34, 39]. In addition, histologic evidence suggests that REPs can support the ingrowth of vital tissue along the root canal system of mature teeth [40]. Moreover, REPs may offer a promising treatment alternative for more complex clinical scenarios, including teeth with root fracture, perforating root resorption, or avulsion [41].

In the present study, a lower concentration of sodium hypochlorite ( NaOCl ) 1.5% was used with side vented needle to avoid apical extrusion of the irrigant into the periapical tissues. Previous studies have demonstrated that high concentrations of NaOCl exert a marked negative effect on the survival and differentiation of stem cells of the apical papilla (SCAPs) [9], mesenchymal stem cells [42], and residual human dental pulp stem cells (hDPSCs) [42]. A final irrigation with 17% EDTA was performed prior to the placement of regenerative endodontic scaffolds, as previous studies have showed its ability to induce the release of bioactive growth factors from the dentine matrix, enhance the survival and expression of stem cells of the apical papilla (SCAPs) and to partially counteract the cytotoxic effects associated with sodium hypochlorite irrigation [9, 43–45].

Ultrasonic activation of EDTA was employed in the present study, as it has been shown to further increase the release of transforming growth factor-beta 1 (TGF-β1) from dentine [46, 47].

In the present study, intracanal disinfection was achieved using a double antibiotic paste (DAP) placed between the appointments, in accordance with the recommendations of the American Association of Endodontics [48], to reduce the risk of tooth discoloration and to limit potential adverse effects on stem cell survival [49]. Evidence from previous investigations demonstrated its effectiveness in reducing microbial load and biofilm formation within infected root canals. Furthermore, it can leave a sustained inhibitory effect on dentin surfaces after removal, which may limit subsequent bacterial recolonization [50, 51].

In the present study, a premixed calcium silicate–based cement (NeoPUTTY) was used as a coronal barrier. Previous work by Hoffman [52] reported a sustained release of calcium ions from NeoPUTTY over a 28-day period, which may be associated with the antimicrobial behavior commonly observed, thereby supporting tissue healing [53].

In this study, CBCT was employed to assess radiographic healing of the periapical region, as it enables detection of small lesions and low-density areas, providing a more accurate estimation of the true lesion size [54, 55]. Furthermore, the use of CBCT-PAI allowed a standardized and reproducible 3D assessment, enhancing lesion detection, follow-up, and clinical decision-making compared with traditional PAI scoring based on periapical radiographs [28].

In addition, standardized conventional two-dimensional radiographs were also used to assess periapical healing. This approach ensured reproducible images with reduced distortion, allowing consistent comparison of lesion size between baseline and follow-up evaluations [56, 57].

The primary outcome, periapical healing, assessed primarily using CBCT and secondarily using CBCT-PAI scoring and two-dimensional radiography, directly reflects treatment success in regenerative endodontic procedures. Clinically, the resolution of periapical pathology is associated with effective infection control, tissue repair, and long-term tooth survival, making it a highly meaningful endpoint for both clinicians and patients.

Additional secondary outcomes, including postoperative pain and clinical normalcy, provide important patient-centered information regarding treatment response. These outcomes are clinically relevant, as they directly influence patient comfort, satisfaction, and overall acceptance of the procedure in routine practice. The combined assessment of objective radiographic healing and patient-reported outcomes allows for a comprehensive evaluation of treatment effectiveness, thereby supporting evidence-based clinical decision-making and enhancing the applicability of regenerative strategies in everyday clinical settings.

Regarding the results, in the present study, all 28 participants were initially recruited and randomly allocated equally between the two groups (allocation ratio 1:1; *n* = 14 per group). Two participants from the control group were not available for the periapical healing assessment. One participant could not be contacted at the 6-month follow-up despite attempts to reach the participant. The second participant reported fracture of the treated tooth before the scheduled 3-month follow-up. Clinical examination revealed fracture of the restoration with loss of part of the tooth structure and the presence of a sinus tract. The case was considered as a treatment failure due to reinfection and was subsequently managed with conventional root canal treatment and referred to the prosthodontic department for definitive restoration.

The available clinical findings suggested bacterial recontamination associated with failure of the coronal restoration; however, because the patient presented before the scheduled radiographic follow-up, no radiographic assessment was available at the time of failure. Therefore, the precise cause of reinfection could not be definitively established, and it cannot be determined whether the event was attributable to coronal microleakage, scaffold-related factors, or other factors associated with the fractured restoration and tooth structure. We have therefore avoided attributing this failure to the bioactivity of the scaffold without direct supporting evidence.

The findings of the present study demonstrated significant periapical lesion reduction over time in both groups, with no statistically significant difference between them, indicating that both scaffolds promoted healing. Postoperative pain profiles were also comparable between groups.

The study groups were comparable at baseline with no significant differences in demographic or clinical characteristics. This supports the adequacy of randomization and strengthens the internal validity of the trial, indicating that observed outcome differences are unlikely to be attributable to baseline imbalance.

Quantitative CBCT analysis revealed significant reductions in lesion area over time in both groups, supporting the notion that platelet concentrates can enhance periapical healing in regenerative endodontics [58, 59]. Although the intervention group exhibited slightly lower postoperative median lesion values, the difference between groups did not reach statistical significance.

This finding is consistent with previous evidence, similar outcomes have been reported in other studies. For instance, Ibrahim et al. [60] observed no significant difference between i-PRF and conventional root canal therapy, Markandey and Das Adhikari [61] reported comparable results among blood clot (BC), platelet-rich plasma (PRP), and platelet-rich fibrin (PRF) when assessed using intraoral periapical radiography and CBCT, and Abo-Heikal et al. [16] found no significant difference between i-PRF and PRP.

CBCT-PAI analysis demonstrated postoperative improvement in periapical status in both groups, with no significant differences between the intervention and control groups. These results are in consistent with previous study which demonstrated a reduction in periapical lesion size when assessed using CBCT-PAI [25].

Although favorable postoperative scores were observed, the lack of significant changes over time within-group comparisons may be explained by the ordinal structure of the CBCT-PAI scoring system. While this index is clinically relevant and widely used, its categorical nature may limit its sensitivity to detect small changes in lesion size when compared with quantitative three-dimensional CBCT measurements and segmentation-based approaches [28, 62]. In regenerative endodontics, several clinical investigations have employed CBCT-based periapical assessment, including CBCT-PAI scoring, to evaluate healing outcomes.

Kateb and Fata [63] assessed regenerative procedures in mature necrotic teeth with baseline CBCT-PAI scores ≥ 3 and reported significant reductions in lesion size after 12 months. Likewise, recent clinical trials and cohort studies have used CBCT-derived quantitative and three-dimensional assessment methods to monitor periapical healing and identify subtle changes that may not be apparent on conventional radiographs [28, 64, 65].

In contrast to CBCT findings, two-dimensional radiographic assessment revealed a statistically significant difference between groups at baseline, with smaller lesion sizes observed in the intervention group. However, this difference was no longer apparent at any postoperative time point, as both groups demonstrated significant lesion reduction over time. The percentage change in 2D lesion area further demonstrated a progressive reduction in lesion size in both groups throughout the follow-up period. Although the intervention group showed a numerically greater reduction at 6, 9, and 12 months, the differences between the groups were not statistically significant. The most notable between-group difference was observed at 9 months, when the intervention group demonstrated a greater mean reduction in lesion area than the control group, accompanied by a moderate effect size (d = 0.73). However, this difference did not reach statistical significance (*P* = 0.077). By 12 months, the difference between the groups was not statistically significant and was associated with a smaller effect size.

These findings suggest that, although the intervention group showed a numerical tendency toward greater lesion reduction at some follow-up points, the overall pattern does not support a statistically significant additional benefit of the intervention over the control treatment in terms of 2D lesion area reduction. Therefore, based on the current data, the intervention cannot be considered superior to the control treatment with respect to 2D lesion area reduction over the 12-month follow-up period.

The discrepancy between baseline two-dimensional and CBCT measurements highlights the inherent limitations of periapical radiographs in accurately representing lesion dimensions, due to anatomical superimposition and projection geometry [28].

Consistent with our findings, significant reductions in periapical lesion diameter were reported when comparing PRF and CGF scaffold on 2D radiographs, with radiographic outcomes consistent with CBCT findings [66]. Similarly, comparable healing outcomes between blood clot and PRF scaffolds have been reported using 2D radiographic assessment [67].

The enhanced regenerative potential observed in the present study can be attributed to, the distinct composition and physical properties i-PRF. Prior to fibrin formation, i-PRF exists as a fluid containing fibrinogen and thrombin, forming a loose and porous network that incorporates numerous biologically active molecules [68]. The relatively gentle centrifugation protocol, shorter spin duration, and use of plain plastic tubes allow for slow physiological polymerization of fibrin, resulting in a flexible clot that functions as a dynamic hydrogel, supports cytokine retention, and better preserves the integrity and quantity of the embedded biomolecule [17, 69].

Furthermore, cross-linking of fibrin fibers enhance the clot stability, facilitates the aggregation of regenerative cells, promotes the formation of microvascular networks, improved cell migration and proliferation, and the promotion of osteogenic differentiation of human mesenchymal stem cells [70], as confirmed by in-vitro studies [71, 72].

In addition, i-PRF contains higher levels of anti-inflammatory cytokines including (IL-4, IL-6, and IL-10), and is enriched with leukocytes, which play a pivotal role in tissue regeneration by modulating various cell types, enhancing angiogenesis and lymphangiogenesis, promoting communication between precursor and mesenchymal cells, and improving platelet functionality [73]. These properties suggested the use of i-PRF in regenerative endodontics [74, 75].

Regarding Vitamin C, is known to contribute to collagen formation and to regulate oxidative stress, both of which are essential factors in establishing a favorable environment for tissue repair and regeneration [76]. These biological effects may support stem cell proliferation, extracellular matrix production, and osteogenic differentiation [77]. Vitamin C has been shown to increase stem cell marker expression in dental cells [78] and can act as an epigenetic regulator of osteogenesis [79], while also supporting stem cell differentiation and matrix synthesis in MSC models [80].

In the present study, a concentration of 250 µM vitamin C was selected based on evidence from several in vitro studies rather than on a pharmacokinetic model specifically established for the root canal environment. Therefore, the 250 µM concentration used in the present study should be regarded as a biologically supported experimental concentration rather than a pharmacokinetically validated concentration. Several in vitro studies reported favorable biological effects at micromolar concentration, that it enhances mesenchymal stem cell proliferation while maintaining phenotype and differentiation potential, making it suitable for regenerative applications. At this concentration, both proliferation and differentiation remain stable, whereas higher concentrations (e.g., 500 µM) have been shown to reduce proliferation and induce morphological changes [81]. Additionally, vitamin C at 250 µM has been reported to promote collagen synthesis, extracellular matrix homeostasis, and stem cell differentiation across multiple cell types [82].

From a tissue engineering perspective, combining antioxidant agents with platelet concentrates is biologically plausible and may enhance regenerative outcomes, supporting the concept of a synergistic interaction between antioxidants and platelet concentrates [83–86]. However, to date no randomized clinical trials have directly compared the adjunctive use of vitamin C with injectable PRF (i-PRF) in regenerative endodontic procedures. Importantly, the magnitude of this potential benefit may not be fully detectable in studies with relatively small sample sizes or limited follow-up periods, and should therefore be interpreted cautiously until further well-designed clinical studies provide additional definitive evidence.

The present study evaluated postoperative pain following regenerative endodontic procedures. Our findings align existing clinical evidence showing that regenerative approaches, including those utilizing platelet concentrates, are generally associated with low levels of early postoperative discomfort. Previous study reported no significant difference in postoperative pain between revascularization and conventional endodontic treatment in mature necrotic single-rooted teeth [40]. Similarly, another study demonstrated no statistically significant differences between regenerative procedure and conventional endodontic treatment, except at 12 h postoperatively, where a significant difference was observed [87]. On the contrary, significantly higher pain levels following revascularization at 6, 12, and 24 h relative to conventional endodontic therapy were reported, which may be attributed to over-instrumentation in the revascularization group [88].

In the present study, the intervention group demonstrated that pain intensity was highest at 6 h postoperatively and decreased thereafter. This early reduction in pain may be attributed to the antioxidant and tissue-healing effects of vitamin C, which can accelerate the resolution of inflammation and enhance tissue recovery. In contrast, the i-PRF alone group did not demonstrate a statistically significant decrease in pain, possibly due to limited modulation of oxidative stress, individual variability. Overall, both regenerative protocols resulted in minimal postoperative discomfort, with the vitamin C-supplemented group exhibiting a slightly faster early decline in pain intensity.

Clinical normalcy was assessed as a secondary outcome in the present investigation. Overall, the absence of clinical symptoms across both groups was consistent with the radiographic findings and supports the favorable clinical performance of the two regenerative approaches.

Previous clinical investigations in regenerative endodontics have reported similar findings, showing that treated teeth remain asymptomatic during follow-up. Success has been primarily defined by the absence of pain, swelling and sinus tract formation, with the majority of cases maintaining functional, symptom-free throughout the observation period. These findings suggest that regenerative protocols provide favorable clinical outcomes and stable resolution of symptoms in the short- to medium-term follow-up [64, 66, 89, 90].

Importantly, the interpretation of these favorable clinical and radiographic findings should be considered in the context of the regenerative protocol used in the present study. Intentional induction of apical bleeding was not performed because instrumentation beyond the anatomical apex could potentially influence the postoperative inflammatory response and pain, which was a predefined secondary outcome. Instead, injectable PRF was used as an alternative intracanal scaffold.

Although periapical healing represents an important clinical outcome of regenerative endodontic procedures, it does not, by itself, confirm true pulp regeneration. Similarly, pulp sensibility responses, when present, should be interpreted cautiously, as a positive response does not necessarily indicate the formation of an organized and functional pulp–dentin complex [91, 92]. Therefore, the clinical and radiographic healing observed in the present study should be interpreted as evidence of treatment response rather than definitive evidence of histologically confirmed pulp regeneration.

A number of methodological features enhanced the internal validity of the present trial. The randomized controlled design with blinded participants and outcome assessors reduced the risk of selection and detection bias. Clinical procedures were standardized across all cases to minimize performance variability, and the use of clinically relevant regenerative scaffolds enhanced the translational value of the findings. Healing outcomes were evaluated using both CBCT and conventional periapical radiography, allowing for a comprehensive assessment of periapical status and tissue response over time. In addition, the inclusion of multiple outcome measures, incorporating both objective radiographic and patient-centered clinical outcomes, further strengthens the comprehensiveness of the study.

In addition, the high level of agreement between independent examiners for CBCT-based quantitative measurements, CBCT-PAI scoring and two- dimensional radiograph demonstrates high reproducibility of the radiographic assessment protocol. Collectively, these methodological features increase confidence that the observed radiographic changes reflect true biological healing rather than measurement variability.

Notwithstanding the strengths of the present investigation, certain limitations should be considered when interpreting the findings. The relatively modest sample size may have reduced the statistical power to detect subtle differences between the treatment groups. Moreover, although the follow-up period was sufficient to assess early healing trends, it may not fully represent long-term regenerative outcomes or the stability of periapical healing over extended periods.

Another limitation is that the present study cannot establish whether the observed clinical and radiographic healing was accompanied by the formation of a functional pulp–dentin complex.

Although increased root wall thickness and/or root length are recognized secondary outcomes of regenerative endodontic procedures, these parameters were not prospectively assessed in the present study because all included teeth had matured, fully developed roots. In addition, pulp sensibility testing was not included in the study protocol; therefore, no sensibility findings were available to provide additional information regarding the presence of vital intracanal tissue. Accordingly, the clinical and radiographic healing observed in the present study should not be interpreted as definitive evidence of true pulp regeneration or restoration of a functional pulp–dentin complex. Future studies incorporating standardized assessment of root wall thickness and root length, pulp sensibility testing and, where feasible, biological or histological evaluation are warranted to further characterize the regenerative response to injectable PRF in mature teeth.

Biological variability inherent to regenerative endodontic procedures should also be acknowledged. Differences in host response, tooth characteristics and the biological behavior of regenerative scaffolds may influence the extent of tissue response and radiographic healing. While treatment protocols were standardized, such variability is difficult to eliminate entirely in clinical settings.

In addition, although outcome assessment was performed under blinded conditions, operator blinding was not feasible due to the nature of the intervention, which may introduce a potential risk of performance bias.

Another limitation of the present study is the absence of a conventional blood clot control group. Accordingly, the present findings cannot determine whether i-PRF, either alone or supplemented with vitamin C, provides superior clinical or radiographic outcomes compared with conventional blood clot–based regenerative endodontic procedures. The findings should therefore be interpreted as a comparison between the two investigated i-PRF formulations, and broader clinical generalization should be made with caution. Future randomized clinical trials directly comparing i-PRF, with or without vitamin C, with conventional blood clot scaffolds and incorporating longer follow-up periods are warranted.

Furthermore, although incorporation of vitamin C into i-PRF may theoretically influence the local retention and release of vitamin C through the fibrin matrix, the release kinetics, oxidation stability, dentinal tubular penetration, and effective duration following its incorporation into i-PRF within root canals remain insufficiently characterized. Future studies should therefore investigate the intracanal concentration time profile of vitamin C/ i-PRF under clinically relevant conditions, such data would provide a stronger scientific basis for determining the optimal concentration and duration of intracanal vitamin C exposure in regenerative endodontics application.

Although two participants from the control group were not available for periapical healing assessment, the final sample of 12 participants remained equal to the prespecified minimum required sample size. Nevertheless, differential loss to follow-up in the control group should be considered when interpreting the periapical healing findings.

Ultimately, the study was carried out within a single clinical center, which may restrict the generalizability of the results to other populations or clinical settings.

Further investigations with larger multicenter cohorts and long-term follow-up periods are warranted to clarify the potential synergistic effects of i-PRF when combined with bioactive molecules such as vitamin C. Future studies should also explore the performance of these regenerative strategies across different tooth types, lesion sizes, and more complex clinical scenarios. Such evidence may help clinicians refine biologically driven regenerative protocols for mature teeth, particularly in cases where conventional scaffolds may offer limited regenerative potential, while maintaining favorable patient-reported outcomes and postoperative comfort.

## Conclusion

Within the limitations of this randomized controlled trial, both injectable platelet-rich fibrin (i-PRF) alone and i-PRF supplemented with vitamin C were associated with favorable clinical and radiographic outcomes when used as scaffolds in regenerative endodontic treatment of necrotic mature teeth with chronic apical periodontitis. No statistically significant differences were observed between the groups with respect to periapical healing, clinical outcomes, or postoperative pain. Both treatment protocols were associated with favorable clinical outcomes, and no adverse events were observed during the follow-up period.

The addition of vitamin C to i-PRF did not demonstrate a statistically significant additional clinical or radiographic benefit under the conditions of the present study. Therefore, the potential added value of vitamin C as an adjunct to i-PRF remains uncertain and warrants further investigation.

Overall, the present findings suggest that both investigated i-PRF protocols may provide favorable clinical and radiographic healing outcomes in necrotic mature teeth. However, the absence of a conventional blood clot control group and the lack of assessment of pulp sensibility and other regenerative parameters limit the ability to draw definitive conclusions regarding true pulp regeneration and the superiority of i-PRF-based scaffolds over conventional regenerative approaches.

## Supplementary Information

Supplementary Material 1.

## Data Availability

The datasets generated and/or analyzed during the current study are available from the corresponding author on reasonable request.

## Abbreviations

RCT: Root canal treatment
REPs: Regenerative endodontic procedures
i-PRF+AA: Injectable platelet rich fibrin with Vitamin C
i-PRF: Injectable platelet rich fibrin
PRP: Platelet rich plasma
PRF: Platelet rich fibrin
DAP: Double antibiotic paste
AAE: American Association of Endodontics
CBCT: Cone beam computed tomography
CBCT-PAI: Cone beam computed tomography- periapical index score
2D: Two dimension
EDTA: Ethylenediaminetetraacetic acid
NaOCl: Sodium hypochlorite
CEJ: Cemento-enamel junction
NRS: Numerical Pain Rating Scale
CONSORT: Consolidated Standards Of Reporting Trials
µM: Micromolar

## References

[1] Yoneda N, Noiri Y, Matsui S, et al. Development of a root canal treatment model in the rat. Sci Rep. 2017;7(1):3315. 10.1038/s41598-017-03628-6. PMC5468248 28607360

[2] Sloan AJ. Biology of the dentin–pulp complex. Stem Cell Biology and Tissue Engineering in Dental Sciences. Elsevier; 2015. pp. 371–8. 10.1016/B978-0-12-397157-9.00033-3.3.

[3] American Association of Endodontists. Clinical considerations for a regenerative procedure. Chicago (IL): American Association of Endodontists; 2022. Available from: https://www.aae.org/specialty/wp-content/uploads/sites/2/2023/04/ConsiderationsForRegEndo_AsOfNov2022-4.pdf. Cited 2026 Aug 22.

[4] Galler K, Krastl G, Simon S, Van Gorp G, Meschi N, et al. European Society of Endodontology position statement: revitalization procedures. Int Endod J. 2016;49(8):717–23. 10.1111/iej.12629. 26990236

[5] Duggal M, Tong HJ, Al-Ansary M, Twati W, Day P, Nazzal H. Interventions for the endodontic management of non-vital traumatised immature permanent anterior teeth in children and adolescents: a systematic review of the evidence and guidelines of the European Academy of Paediatric Dentistry. Eur Arch Paediatr Dent. 2017;18(3):139–51. 10.1007/s40368-017-0289-5. PMC5488080 28508244

[6] Östby BN. The role of the blood clot in endodontic therapy: an experimental histologic study. Acta Odontol Scand. 1961;19(3–4):323–53. 10.3109/00016356109043395. 14482575

[7] Kontakiotis EG, Filippatos CG, Tzanetakis GN, Agrafioti A. Regenerative endodontic therapy: a data analysis of clinical protocols. J Endod. 2015;41(2):146–54. 10.1016/j.joen.2014.08.003. 25301351

[8] Nazzal H, Tong HJ, Nixon PJ, Duggal M. Regenerative endodontic therapy for managing immature non-vital teeth: a national survey of UK paediatric dental specialists and trainees. Br Dent J. 2018;224(4):247–54. 10.1038/sj.bdj.2018.122. 29472688

[9] Martin DE, De Almeida JFA, Henry MA, et al. Concentration-dependent effect of sodium hypochlorite on stem cells of apical papilla survival and differentiation. J Endod. 2014;40(1):51–5. 10.1016/j.joen.2013.07.026. 24331991

[10] Nagata JY, De Almeida Gomes BPF, Lima TFR, et al. Traumatized immature teeth treated with two protocols of pulp revascularization. J Endod. 2014;40(5):606–12. 10.1016/j.joen.2014.01.032. 24767551

[11] Kim SG, Malek M, Sigurdsson A, Lin LM, Kahler B. Regenerative endodontics: a comprehensive review. Int Endod J. 2018;51(12):1367–88. 10.1111/iej.12954. 29777616

[12] Paryani K, Kim SG. Regenerative endodontic treatment of permanent teeth after completion of root development: a report of two cases. J Endod. 2013;39(7):929–34. 10.1016/j.joen.2013.04.029. 23791266

[13] Ríos-Osorio N, Caviedes-Bucheli J, Jimenez-Peña O, et al. Comparative outcomes of platelet concentrates and blood clot scaffolds for regenerative endodontic procedures: a systematic review of randomized controlled clinical trials. J Clin Exp Dent. 2023;15(3):e239–49. 10.4317/jced.60150. PMC10062460 37008236

[14] Gomes-Filho JE, Duarte PCT, Ervolino E, Bomfim SRM, Abimussi CJX, et al. Histologic characterization of engineered tissues in the canal space of closed-apex teeth with apical periodontitis. J Endod. 2013;39(12):1549–56. 10.1016/j.joen.2013.08.023. 24238445

[15] Choukroun J, Adda F, Schoeffler C, Vervelle A. Une opportunité en paro-implantologie: le PRF. Implantodontie. 2001;42:55–62.

[16] Abo-Heikal MM, El-Shafei JM, Shouman SA, Roshdy NN. Evaluation of the efficacy of injectable platelet-rich fibrin versus platelet-rich plasma in the regeneration of traumatized necrotic immature maxillary anterior teeth: a randomized clinical trial. Dent Traumatol. 2024;40(1):61–75. 10.1111/edt.12881. 37612879

[17] Choukroun J, Ghanaati S. Reduction of relative centrifugation force within injectable platelet-rich fibrin (PRF) concentrates advances patients’ own inflammatory cells, platelets and growth factors: the first introduction to the low-speed centrifugation concept. Eur J Trauma Emerg Surg. 2018;44(1):87–95. 10.1007/s00068-017-0767-9. PMC5808086 28283682

[18] Kyyak S, Blatt S, Pabst A, Thiem D, Al-Nawas B, Kämmerer PW. Combination of an allogenic and a xenogenic bone substitute material with injectable platelet-rich fibrin: a comparative in vitro study. J Biomater Appl. 2020;35(1):83–96. 10.1177/088532822091440719. 32237950

[19] Farshidfar N, Amiri MA, Estrin NE, Ahmad P, Sculean A, et al. Platelet-rich plasma (PRP) versus injectable platelet-rich fibrin (i-PRF): a systematic review across all fields of medicine. Periodontol 2000. 2025. 10.1111/prd.12626.20. PMC13428083 40125556

[20] Chai J, Jin R, Yuan G, Kanter V, Miron RJ, Zhang Y. Effect of liquid platelet-rich fibrin and platelet-rich plasma on the regenerative potential of dental pulp cells cultured under inflammatory conditions: a comparative analysis. J Endod. 2019;45(8):1000–8. 10.1016/j.joen.2019.04.002. 31248700

[21] Zhang J, Yin C, Zhao Q, et al. Anti-inflammation effects of injectable platelet-rich fibrin via macrophages and dendritic cells. J Biomed Mater Res A. 2020;108(1):61–8. 10.1002/jbm.a.36792. 31449340

[22] Li Y, Zhang W, Chang L, et al. Vitamin C alleviates aging defects in a stem cell model for Werner syndrome. Protein Cell. 2016;7(7):478–88. 10.1007/s13238-016-0278-1. PMC4930768 27271327

[23] Urban K, Höhling HJ, Lüttenberg B, Szuwart T, Plate U. An in vitro study of osteoblast vitality influenced by the vitamins C and E. Head Face Med. 2012;8:25. 10.1186/1746-160X-8-25. PMC3489720 23021517

[24] Zhang P, Li J, Qi Y, et al. Vitamin C promotes the proliferation of human adipose-derived stem cells via the p53–p21 pathway. Organogenesis. 2016;12(3):143–51. 10.1080/15476278.2016.1194148. PMC4993295 27231022

[25] Elsheshtawy A, Nazzal H, El Shahawy O, El Baz A, Ismail S, et al. The effect of platelet-rich plasma as a scaffold in regeneration/revitalization endodontics of immature permanent teeth assessed using two-dimensional radiographs and cone beam computed tomography: a randomized controlled trial. Int Endod J. 2020;53(7):905–21. 10.1111/iej.13303. 32249441

[26] Nageh M, Ibrahim LA, Abunaeem FM, Salam E. Management of internal inflammatory root resorption using injectable platelet-rich fibrin revascularization technique: a clinical study with cone-beam computed tomography evaluation. Clin Oral Investig. 2022;26(2):1505–16. 10.1007/s00784-021-04123-z. 34387731

[27] Van Pham P, Tran NY, Phan NLC, Vu NB, Phan NK. Vitamin C stimulates human gingival stem cell proliferation and expression of pluripotent markers. Vitro Cell Dev Biol Anim. 2016;52(2):218–27. 10.1007/s11626-015-9963-2. 26487430

[28] Estrela C, Bueno MR, Azevedo BC, Azevedo JR, Pécora JD. A new periapical index based on cone beam computed tomography. J Endod. 2008;34(11):1325–31. 10.1016/j.joen.2008.08.013. 18928840

[29] Bose R, Nummikoski P, Hargreaves K. A retrospective evaluation of radiographic outcomes in immature teeth with necrotic root canal systems treated with regenerative endodontic procedures. J Endod. 2009;35(10):1343–9. 10.1016/j.joen.2009.06.021. 19801227

[30] Flake NM, Gibbs JL, Diogenes A, Hargreaves KM, Khan AA. A standardized novel method to measure radiographic root changes after endodontic therapy in immature teeth. J Endod. 2014;40(1):46–50. 10.1016/j.joen.2013.09.025. PMC3922299 24331990

[31] Williamson A, Hoggart B. Pain: a review of three commonly used pain rating scales. J Clin Nurs. 2005;14(7):798–804. 10.1111/j.1365-2702.2005.01121.x. 16000093

[32] Morse DR. Age-related changes of the dental pulp complex and their relationship to systemic aging. Oral Surg Oral Med Oral Pathol. 1991;72(6):721–45. 10.1016/0030-4220(91)90019-9. 1812456

[33] Ng YL, Mann V, Gulabivala K. Outcome of secondary root canal treatment: a systematic review of the literature. Int Endod J. 2008;41(12):1026–46. 10.1111/j.1365-2591.2008.01484.x. 19133093

[34] Saoud TMA, Martin G, Chen YHM, et al. Treatment of mature permanent teeth with necrotic pulps and apical periodontitis using regenerative endodontic procedures: a case series. J Endod. 2016;42(1):57–65. 10.1016/j.joen.2015.09.015. 26525552

[35] Anna D, Dimitra S, Kleoniki L. Regenerative procedures in mature teeth: a new era in endodontics? A systematic review. Int J Dent Oral Health. 2020;6(4):1–13.

[36] Glynis A, Foschi F, Kefalou I, Koletsi D, Tzanetakis GN. Regenerative endodontic procedures for the treatment of necrotic mature teeth with apical periodontitis: a systematic review and meta-analysis of randomized controlled trials. J Endod. 2021;47(6):873–82. 10.1016/j.joen.2021.03.015. 33811981

[37] Nageh M, Ahmed GM, El-Baz AA. Assessment of regaining pulp sensibility in mature necrotic teeth using a modified revascularization technique with platelet-rich fibrin: a clinical study. J Endod. 2018;44(10):1526–33. 10.1016/j.joen.2018.06.014. 30174103

[38] Dammaschke T, Steven D, Kaup M, Ott KHR. Long-term survival of root canal-treated teeth: a retrospective study over 10 years. J Endod. 2003;29(10):638–43. 10.1097/00004770-200310000-00006. 14606785

[39] Abou Samra RA, El Backly RM, Aly HM, Nouh SR, Moussa SM. Revascularization in mature permanent teeth with necrotic pulp and apical periodontitis: case series. Alex Dent J. 2018;43(2):7–12. 10.21608/adjalexu.2018.57617.

[40] Arslan H, Ahmed HMA, Şahin Y, Yıldız ED, Gündoğdu EC, et al. Regenerative endodontic procedures in necrotic mature teeth with periapical radiolucencies: a preliminary randomized clinical study. J Endod. 2019;45(7):863–72. 10.1016/j.joen.2019.04.005. 31155298

[41] Saoud TMA, Mistry S, Kahler B, Sigurdsson A, Lin LM. Regenerative endodontic procedures for traumatized teeth after horizontal root fracture, avulsion and perforating root resorption. J Endod. 2016;42(10):1476–82. 10.1016/j.joen.2016.04.028. 27576211

[42] Li Y, Jin C, Zhao S, Xie H. Effects of sodium hypochlorite and ethylenediaminetetraacetic acid on proliferation, osteogenic/odontogenic differentiation and mechanosensitive gene expression of human dental pulp stem cells. Tissue Cell. 2022;79:101955. 10.1016/j.tice.2022.101955. 36265367

[43] Yamauchi N, Nagaoka H, Yamauchi S, Teixeira FB, Miguez P, Yamauchi M. Immunohistological characterization of newly formed tissues after regenerative procedure in immature dog teeth. J Endod. 2011;37(12):1636–41. 10.1016/j.joen.2011.08.025. 22099896

[44] Galler KM, Buchalla W, Hiller KA, Federlin M, Eidt A, et al. Influence of root canal disinfectants on growth factor release from dentin. J Endod. 2015;41(3):363–8. 10.1016/j.joen.2014.11.021. 25595468

[45] Kawamura R, Hayashi Y, Murakami H, Nakashima M. EDTA soluble chemical components and the conditioned medium from mobilized dental pulp stem cells contain an inductive microenvironment promoting cell proliferation, migration and odontoblastic differentiation. Stem Cell Res Ther. 2016;7(1):77. 10.1186/s13287-016-0334-z. PMC4937592 27387974

[46] Widbiller M, Eidt A, Hiller KA, Buchalla W, Schmalz G, Galler K. Ultrasonic activation of irrigants increases growth factor release from human dentine. Clin Oral Investig. 2017;21(3):879–88. 10.1007/s00784-016-1824-1. 27114090

[47] Aksel H, Albanyan H, Bosaid F, Azim AA. Dentin conditioning protocol for regenerative endodontic procedures. J Endod. 2020;46(8):1099–104. 10.1016/j.joen.2020.05.010. 32479803

[48] American Association of Endodontists. Clinical considerations for a regenerative procedure. 2016. Available from: https://aae.org/specialty/wpcontent/uploads/sites/2/2017/06/currentregenerativeendodonticconsiderations.pdf. Cited 2026 Mar 10.

[49] Kahler B, Rossi-Fedele G. A review of tooth discoloration after regenerative endodontic therapy. J Endod. 2016;42(4):563–9. 10.1016/j.joen.2015.12.022. 26852148

[50] Sabrah AH, Yassen GH, Gregory RL. Effectiveness of antibiotic medicaments against biofilm formation of Enterococcus faecalis and Porphyromonas gingivalis. J Endod. 2013;39(11):1385–9. 10.1016/j.joen.2013.05.003. 24139259

[51] Jenks DB, Ehrlich Y, Spolnik K, Gregory RL, Yassen GH. Residual antibiofilm effects of various concentrations of double antibiotic paste used during regenerative endodontics after different application times. Arch Oral Biol. 2016;70:88–93. 10.1016/j.archoralbio.2016.06.006. 27341460

[52] Hoffman Y. Shear bond strength comparison of bioceramics to root dentin: PR09. J Endod. 2023;49(5):e15. 10.33915/etd.12008.

[53] Lozano-Guillén A, López-García S, Rodríguez-Lozano FJ, Sanz JL, Lozano A, et al. Comparative cytocompatibility of the new calcium silicate-based cement NeoPutty versus NeoMTA Plus and MTA on human dental pulp cells: an in vitro study. Clin Oral Investig. 2022;26(12):7219–28. 10.1007/s00784-022-04682-9. PMC9708762 36053352

[54] Ahlowalia M, Patel S, Anwar H, Cama G, Austin R, Wilson R, et al. Accuracy of CBCT for volumetric measurement of simulated periapical lesions. Int Endod J. 2013;46(6):538–46. 10.1111/iej.12023. 23216253

[55] Shetty H, Shetty S, Kakade A, Mali S, Shetty A, Neelakantan P. Three-dimensional qualitative and quantitative analyses of the effect of periradicular lesions on the outcome of regenerative endodontic procedures: a prospective clinical study. Clin Oral Investig. 2021;25(2):691–700. 10.1007/s00784-020-03583-z. 32954475

[56] Sadek SA, Abbas HM, Alfelali M, Almahdali A. Using acrylic customized X-ray positioning stents for long-term follow-up studies. Saudi Dent J. 2020;32(3):120–8. 10.1016/j.sdentj.2019.10.001. PMC7063427 32180668

[57] Youssef A, Ali M, ElBolok A, Hassan R. Regenerative endodontic procedures for the treatment of necrotic mature teeth: a preliminary randomized clinical trial. Int Endod J. 2022;55(4):334–46. 10.1111/iej.13681. 35030270

[58] Huidobro-Guerrero AA, Santoy-Lozano A, Lopez-Martinez F, Garcia-Jau RA, Valle-Urias AE, et al. Autologous platelet concentrates in regenerative endodontic treatment. Int J Appl Dent Sci. 2021;7(4):20–4. 10.22271/oral.2021.v7.i4a.1347.

[59] Yoshpe M, Kaufman A, Lin S, et al. Regenerative endodontics: a promising tool to promote periapical healing and root maturation of necrotic immature permanent molars with apical periodontitis using platelet-rich fibrin (PRF). Eur Arch Paediatr Dent. 2021;22(3):527–34. 10.1007/s40368-020-00572-4. 33111235

[60] Ibrahim L, Tawfik M, Abu Naeem F. Evaluation of the periapical healing following pulp revascularization using injectable PRF versus nonsurgical root canal treatment in mature permanent teeth with periapical periodontitis: a clinical study. Egypt Dent J. 2021;67(3):2663–72. 10.21608/edj.2021.62271.1521.

[61] Markandey S, Das Adhikari H. Evaluation of blood clot, platelet-rich plasma and platelet-rich fibrin-mediated regenerative endodontic procedures in teeth with periapical pathology: a CBCT study. Restor Dent Endod. 2022;47(4):e41. 10.5395/rde.2022.47.e41. PMC9715374 36518608

[62] Boubaris M, Chan KL, Zhao W, Cameron A, Sun J, et al. A novel volume-based cone-beam computed tomographic periapical index. J Endod. 2021;47(8):1308–13. 10.1016/j.joen.2021.04.027. 33984376

[63] El Kateb NM, Fata MM. Influence of periapical lesion size on healing outcome following regenerative endodontic procedures: a clinical investigation. Oral Radiol. 2022;38(4):480–9. 10.1007/s11282-021-00578-8. 34826060

[64] Elheeny AAH, Hussien OSE, Abdelmotelb MA, Elmakawi YM, Wahba NKO. A 12-month randomized controlled trial to assess the efficacy of revitalization of retreated mature incisors with periapical radiolucency in adolescents. Sci Rep. 2024;14(1):16366. 10.1038/s41598-024-66305-5. PMC11649900 39013938

[65] Baraka M, El-Kateb N, Gamal M, Elwan A, Sharaf P, et al. Assessment of volumetric changes after regenerative endodontic procedures using semiautomated and 3D U-Net automated CBCT segmentation: a retrospective cohort study. BMC Oral Health. 2025;25(1):1409. 10.1186/s12903-025-06826-1. PMC12442267 40958117

[66] Elheeny AAH, Tony GE. Two-dimensional radiographs and cone-beam computed tomography assessment of concentrated growth factor and platelet-rich fibrin scaffolds in regenerative endodontic treatment of immature incisors with periapical radiolucency: a randomized clinical trial. J Endod. 2024;50(6):792–806. 10.1016/j.joen.2024.01.017. 38281658

[67] Thakkar S, Naik S, Nadig B, Bellal S, Thaliyil A. A comparative clinico-radiographic analysis of regenerative endodontic procedure on immature necrotic permanent teeth using blood clot and PRF as scaffold: a retrospective study. Saudi Dent J. 2023;35(6):753–9. 10.1016/j.sdentj.2023.05.026. PMC10562131 37823081

[68] Jasmine S, Thangavelu A, Krishnamoorthy R, Alzahrani KE, Alshuniaber MA. Architectural and ultrastructural variations of human leukocyte-rich platelet-rich fibrin and injectable platelet-rich fibrin. J Microsc Ultrastruct. 2021;9(2):76–80. 10.4103/JMAU.JMAU_7_20. PMC8291098 34350103

[69] Varela HA, Souza JC, Nascimento RM, et al. Injectable platelet-rich fibrin: cell content, morphological and protein characterization. Clin Oral Investig. 2019;23(3):1309–18. 10.1007/s00784-018-2555-2. 30003342

[70] Dohan Ehrenfest DM, Bielecki T, Jimbo R, Barbé G, Del Corso M, et al. Do the fibrin architecture and leukocyte content influence the growth factor release of platelet concentrates? An evidence-based answer comparing a pure platelet-rich plasma (P-PRP) gel and a leukocyte- and platelet-rich fibrin (L-PRF). Curr Pharm Biotechnol. 2012;13(7):1145–52. 10.2174/138920112800624382. 21740377

[71] Iozon S, Caracostea GV, Páll E, Şoriţău O, Mănăloiu ID, et al. Injectable platelet-rich fibrin influences the behavior of gingival mesenchymal stem cells. Rom J Morphol Embryol. 2020;61(1):189–98. 10.47162/RJME.61.1.21. PMC7728122 32747910

[72] Farshidfar N, Amiri MA, Jafarpour D, Hamedani S, Niknezhad SV, Tayebi L. The feasibility of injectable PRF (I-PRF) for bone tissue engineering and its application in oral and maxillofacial reconstruction: from bench to chairside. Biomater Adv. 2022;134:112557. 10.1016/j.msec.2021.112557. PMC9295636 35527147

[73] Jasmine S, Thangavelu A, Krishnamoorthy R, Alshuniaber MA, Alshatwi AA. Cytokine expression pattern and protein–protein interaction network analysis of leucocyte-rich platelet-rich fibrin and injectable form of platelet-rich fibrin. Oral Maxillofac Surg. 2021;25(2):223–9. 10.1007/s10006-020-00899-8. 32915342

[74] Miron RJ, Fujioka-Kobayashi M, Bishara M, Zhang Y, Hernandez M, Choukroun J. Platelet-rich fibrin and soft tissue wound healing: a systematic review. Tissue Eng Part B Rev. 2017;23(1):83–99. 10.1089/ten.teb.2016.0233. 27672729

[75] Fujioka-Kobayashi M, Miron RJ, Hernandez M, Kandalam U, Zhang Y, Choukroun J. Optimized platelet-rich fibrin with the low-speed concept: growth factor release, biocompatibility and cellular response. J Periodontol. 2017;88(1):112–21. 10.1902/jop.2016.160443. 27587367

[76] DePhillipo NN, Aman ZS, Kennedy MI, Begley J, Moatshe G, et al. Efficacy of vitamin C supplementation on collagen synthesis and oxidative stress after musculoskeletal injuries: a systematic review. Orthop J Sports Med. 2018;6(10):2325967118804544. 10.1177/2325967118804544. PMC6204628 30386805

[77] Salem A, El-Ghlban S, Montaser A, Abdelhameed MF, Attia MF. Enhanced wound healing, anti-inflammatory, and anti-oxidative effects of vitamin C-loaded hyaluronic acid–collagen scaffolds in preclinical rat models. ACS Biomater Sci Eng. 2025;11(12):7461–73. 10.1021/acsbiomaterials.5c01601. 41238212

[78] Diederich A, Fründ HJ, Trojanowicz B, Navarrete Santos A, Nguyen AD, et al. Influence of ascorbic acid as a growth and differentiation factor on dental stem cells used in regenerative endodontic therapies. J Clin Med. 2023;12(3):1196. 10.3390/jcm12031196. PMC9917775 36769844

[79] Thaler R. Vitamin C as an epigenetic gatekeeper of osteogenesis and skeletal health: a new perspective on an ancient molecule. J Bone Min Res. 2025;41(4):353–65. 10.1093/jbmr/zjaf174. PMC13034669 41269246

[80] Bhandi S, Alkahtani A, Mashyakhy M, et al. Effect of ascorbic acid on differentiation, secretome and stemness of stem cells from human exfoliated deciduous tooth (SHEDs). J Pers Med. 2021;11(7):589. 10.3390/jpm11070589. PMC8304986 34206203

[81] Kumar Mekala N, Raju Baadhe R, Rao Parcha S, Devi YP. Enhanced proliferation and osteogenic differentiation of human umbilical cord blood stem cells by L-ascorbic acid, in vitro. Curr Stem Cell Res Ther. 2013;8(2):156–62. 10.2174/157488813805219758. 23140501

[82] D’Anniello C, Cermola F, Patriarca EJ, Minchiotti G. Vitamin C in stem cell biology: impact on extracellular matrix homeostasis and epigenetics. Stem Cells Int. 2017;2017:8936156. 10.1155/2017/8936156. PMC5415867 28512473

[83] Dohle E, El Bagdadi K, Sader R, Choukroun J, Kirkpatrick CJ, Ghanaati S. Platelet-rich fibrin-based matrices to improve angiogenesis in an in vitro co-culture model for bone tissue engineering. J Tissue Eng Regen Med. 2018;12(3):598–610. 10.1002/term.2475. PMC5888144 28509340

[84] Abdulhameed EA, Rani KA, Alghalban FM, Abou Neel EA, Khalifa N, Khalil KA, et al. Managing oxidative stress using vitamin C to improve biocompatibility of polycaprolactone for bone regeneration in vitro. ACS Omega. 2024;9(29):31776–88. 10.1021/acsomega.4c04472. PMC11270701 39072128

[85] Kulkarni M, Sowmya N, Gayathri G, Triveni M. Effects of ascorbic acid augmented albumin platelet-rich fibrin on the wound healing activity of human gingival fibroblasts: an in vitro trial. J Korean Assoc Oral Maxillofac Surg. 2024;50(4):206–15. 10.5125/jkaoms.2024.50.4.206. PMC11372228 39211969

[86] Ozcan M, Topcu BE, Alkaya B, Yilmaz MB, Turer OU, Haytac MC. Effects of ascorbic acid and cholecalciferol on growth factor release, cytokines, and biomechanical properties of platelet rich fibrin: in vitro study. BMC Oral Health. 2026;26(1):262. 10.1186/s12903-025-07620-9. PMC12888708 41527062

[87] Ahmed YE, Ahmed GM, Ghoneim AG. Evaluation of postoperative pain and healing following regenerative endodontics using platelet-rich plasma versus conventional endodontic treatment in necrotic mature mandibular molars with chronic periapical periodontitis: a randomized clinical trial. Int Endod J. 2023;56(4):404–18. 10.1111/iej.13886. 36565044

[88] Tawfik MN, Morsi DM, Ibrahim LA. A comparative study to assess the postoperative pain intensity following non-surgical root canal treatment and pulp revascularization in mature necrotic teeth. Egypt Dent J. 2019;65(4):3899–905. 10.21608/edj.2019.76037.

[89] Liang Y, Ma R, Chen L, et al. Efficacy of i-PRF in regenerative endodontics therapy for mature permanent teeth with pulp necrosis: study protocol for a multicentre randomised controlled trial. Trials. 2021;22(1):436. 10.1186/s13063-021-05401-7. PMC8261915 34229752

[90] Alfahadi HR, Al-Nazhan S, Alkazman FH, Al-Maflehi N, Al-Nazhan N. Clinical and radiographic outcomes of regenerative endodontic treatment performed by endodontic postgraduate students: a retrospective study. Restor Dent Endod. 2022;47(2):e24. 10.5395/rde.2022.47.e24. PMC9160761 35692225

[91] El-Kateb NM, El-Backly RN, Amin WM, Abdalla AM. Quantitative Assessment of Intracanal Regenerated Tissues after Regenerative Endodontic Procedures in Mature Teeth Using Magnetic Resonance Imaging: A Randomized Controlled Clinical Trial. J Endod. 2020;46(5):563–74. 10.1016/j.joen.2020.01.026. 32173020

[92] Widbiller M, Schmalz G. Endodontic regeneration: hard shell, soft core. Odontology. 2021;109(2):303–12. 10.1007/s10266-020-00573-1. PMC7954765 33263826

[93] Ahmad ZH, Alkahtany SM, Anil S. An in vitro evaluation of the cytotoxicity of varying concentrations of sodium hypochlorite on human mesenchymal stem cells. J Contemp Dent Pract. 2014;15(4):473–81. 10.5005/jp-journals-10024-1565. 25576116

